# Composition of the Intranuclear Inclusions of Fragile X-associated Tremor/Ataxia Syndrome

**DOI:** 10.1186/s40478-019-0796-1

**Published:** 2019-09-03

**Authors:** Lisa Ma, Anthony W. Herren, Glenda Espinal, Jamie Randol, Bridget McLaughlin, Veronica Martinez-Cerdeño, Isaac N. Pessah, Randi J. Hagerman, Paul J. Hagerman

**Affiliations:** 1 Department of Biochemistry and Molecular Medicine, University of California Davis, School of Medicine, One Shields Ave, Davis, CA USA; 2 Genome Center, University of California Davis, Davis, California, USA; 3 Department of Pathology and Laboratory Medicine, University of California Davis, School of Medicine, Sacramento, California, USA; 4 Institute for Pediatric Regenerative Medicine, Shriners Hospital of Northern California, University of California Davis, School of Medicine, Sacramento, California, USA; 5 MIND Institute, University of California Davis Health, Sacramento, California, USA; 6 Department of Molecular Biosciences, University of California Davis, School of Veterinary Medicine, Davis, California, USA; 7 Department of Pediatrics, University of California Davis, School of Medicine, Sacramento, California, USA

**Keywords:** Fragile X, neurodegeneration, proteomics, CGG repeat, proteasome, inclusion, FXTAS, FMRpolyG, SUMO, ubiquitin

## Abstract

Fragile X-associated tremor/ataxia syndrome (FXTAS) is a neurodegenerative disorder associated with a premutation repeat expansion (55–200 CGG repeats) in the 5′ noncoding region of the *FMR1* gene. Solitary intranuclear inclusions within FXTAS neurons and astrocytes constitute a hallmark of the disorder, yet our understanding of how and why these bodies form is limited. Here, we have discovered that FXTAS inclusions emit a distinct autofluorescence spectrum, which forms the basis of a novel, unbiased method for isolating FXTAS inclusions by preparative fluorescence-activated cell sorting (FACS). Using a combination of autofluorescence-based FACS and liquid chromatography/tandem mass spectrometry (LC-MS/MS)-based proteomics, we have identified more than two hundred proteins that are enriched within the inclusions relative to FXTAS whole nuclei. Whereas no single protein species dominates inclusion composition, highly enriched levels of conjugated small ubiquitin-related modifier 2 (SUMO 2) protein and p62/sequestosome-1 (p62/SQSTM1) protein were found within the inclusions. Many additional proteins involved with RNA binding, protein turnover, and DNA damage repair were enriched within inclusions relative to total nuclear protein. The current analysis has also allowed the first direct detection, through peptide sequencing, of endogenous FMRpolyG peptide, the product of repeat-associated non-ATG (RAN) translation of the *FMR1* mRNA. However, this peptide was found only at extremely low levels and not within whole FXTAS nuclear preparations, raising the question whether endogenous RAN products exist at quantities sufficient to contribute to FXTAS pathogenesis. The abundance of the inclusion-associated ubiquitin- and SUMO-based modifiers supports a model for inclusion formation as the result of increased protein loads and elevated oxidative stress leading to maladaptive autophagy. These results highlight the need to further investigate FXTAS pathogenesis in the context of endogenous systems.

**Electronic supplementary material:**

The online version of this article (10.1186/s40478-019-0796-1) contains supplementary material, which is available to authorized users.

## Introduction

Fragile X-associated tremor/ataxia syndrome (FXTAS) is a progressive X-linked neurodegenerative disorder that arises from premutation CGG-repeat expansions (55-200 repeats) in the 5′ noncoding portion of the *FMR1* gene [42]. The disorder, which generally has a clinical onset after age fifty, has core features of progressive cerebellar gait ataxia and kinetic tremor, with associated features of executive dysfunction, cognitive decline, neuropathy, dysautonomia, and Parkinsonism [14, 42, 45, 46]. Neuropathologic features of FXTAS include prominent white matter disease, loss of brain volume, Purkinje cell dropout, and solitary ubiquitin-positive inclusions within the nuclei of neurons and astrocytes [42, 44]. Inclusion formation is favored in cortical and hippocampal neurons and astrocytes and can be found in 2–20% of these cells in many patients [38]. Although many neurodegenerative diseases form inclusions and other aggregates in brain tissue, almost all of them include cytoplasmic aggregate formation and form multiple inclusions per cell [3, 7, 11, 17, 59]. Although FXTAS inclusions are found almost exclusively as solitary, spherical particles – distinct from nucleoli – within each nucleus, twinning of inclusions has been reported [5, 38, 39]. The mechanism(s) governing inclusion formation, and the nature of their composition, remain largely unknown; a better understanding of the properties of inclusions is likely to be key in understanding FXTAS pathogenesis.

FXTAS is largely limited to the premutation range, where there is normal to increased transcription of the expanded CGG-repeat mRNA [42, 68, 134]. The absence of the neurodegenerative phenotype for alleles in the full mutation range (>200 CGG repeats), with rare exceptions among mosaics [55, 82, 114], is thought to be due to methylation-coupled transcriptional silencing of the *FMR1* gene. The requirement for transcriptional activity supports an RNA gain-of-function toxicity model [43, 78], as described earlier for myotonic dystrophy (DM) [25, 30, 89, 136].

Several specific models have been proposed to explain how neurotoxicity arises from the expanded CGG-repeat mRNA [118]. Analogous to the model for DM [136], the FXTAS mRNA sequestration model posits that the expanded-repeat *FMR1* mRNA binds excessive amounts of one or more RNA-binding proteins [60, 115, 126, 127, 130], thus rendering those proteins functionally depleted. A second model proposes that initiation of translation at a non-AUG codon upstream of the CGG repeat generates an out-of-frame, toxic FMRpolyG protein [35, 125, 137]. Several sub-mechanisms are related to the FMRpolyG mechanism, including its inhibition of the ubiquitin-proteasomal system [53, 104] and co-aggregation with the nuclear lamina-associated polypeptide 2 beta (LAP2β) [125] or the splicing regulator transformer-2 protein homolog alpha (TRA2A) [19]. Previous studies have also found evidence for mediators of a DNA damage response (DDR) in both mouse and human tissues [44, 54, 58, 117]. However, the data of Robin et al. [117] suggest that the DDR may be a late response to early-onset calcium dysregulation and oxidative stress in affected neurons, progressing through the course of FXTAS pathogenesis. In support of this hypothesis, the *FMR1* premutation model exhibits functional abnormalities early in development [16, 18, 23, 64, 81]. In all of these models, the role of inclusion formation/composition is of central importance, particularly with respect to its role as a repository for the products of aggregation/co-aggregation and to its use as a target for antibody staining to identify the proteins involved with FXTAS pathogenesis. Accordingly, determining both the general composition of the FXTAS inclusions and the estimated relative abundances of the component proteins is of critical importance.

Due to the technical challenges associated with purifying the FXTAS inclusions, their composition has been difficult to ascertain. Furthermore, the reliance on immunofluorescence methods to probe inclusion composition, and to direct their isolation, as in Iwahashi et al. [58], can introduce substantial bias because of the potential for antibody cross-reactivity and variability of the accessibility of their protein targets. In our efforts to address this issue, we have now observed that the FXTAS inclusions emit considerable autofluorescence across a broad range of wavelengths. By coupling FXTAS inclusion autofluorescence with fluorescence-activated cell sorting (FACS), we have been able to exploit this intrinsic property for rapid isolation of the inclusions for downstream characterization. We have coupled this unbiased autofluorescence-based isolation method with mass spectrometry (MS)-based proteomics to characterize the protein composition of FXTAS inclusions. Although using autofluorescence for sorting intracellular aggregates is new in the field of neurodegenerative disease, similar strategies have already been successfully applied in other fields [119, 129, 147]. This analysis allows us to estimate the relative abundances of certain proteins within FXTAS inclusions without the potential confounders of autofluorescence or antibody cross-reactivity, thus aiding the evaluation of existing models for FXTAS pathogenesis.

In the current work, we show that FXTAS inclusions are composed principally of protein and that, of the nearly 200 proteins that are enriched in the inclusions, over half are involved in RNA binding and/or protein turnover. In particular, heterogeneous nuclear ribonucleoproteins (hnRNPs), molecular chaperones, and protein modifiers are prevalent. No single protein species dominates the collection of enriched proteins. FMRpolyG is not detected in the FXTAS nuclei and is only detected at <0.05% molar abundance in the inclusions themselves. Among the most highly abundant of the enriched proteins are the small ubiquitin-related modifier 2 (SUMO 2), ubiquitin, and p62/sequestosome-1 (p62/SQSTM1; hereafter designated p62). Western blot and immunofluorescence experiments confirm that conjugated SUMO 2 is present at over 10 times higher levels in FXTAS patient brain nuclei compared with control brain nuclei and exists primarily in nuclear aggregates. SUMO 2 immunoprecipitation (IP) proteomics demonstrate that conjugated SUMO 2 in FXTAS samples is not due to conjugation to any one specific protein, but an overall higher level of conjugation to numerous proteins, specifically to DDR mediators and proteins involved in cellular response to oxidative stress. These results indicate that inclusions are mainly accumulations of proteins destined for removal that may have aggregated in response to the presence of RNA and/or exceeded the capacity of the nuclear proteasomal machinery. We suggest that once the size/abundance of the aggregates exceeds the threshold of proteasomal degradation, the continued aggregation leads to inclusion formation. For non-dividing cells, p62-directed autophagy is not available to clear the large nuclear aggregates, culminating in the trapped inclusion mass that is a hallmark feature of FXTAS.

## Materials and Methods

### Patient sample information and tissue preparation

Human postmortem frontal cortex samples from six FXTAS, one fragile X syndrome (FXS), one amyotrophic lateral sclerosis (ALS), one Parkinson’s disease (PD), and three control brains were obtained from the FXTAS brain repository at the University of California, Davis (UCD), School of Medicine. Additional human postmortem frontal cortex samples from two Alzheimer’s disease (AD), one frontotemporal dementia (FTD), two Huntington’s Disease (HD), and two progressive supranuclear palsy (PSP) cases were obtained from the NIH Brain & Tissue Repository. Human postmortem frontal cortex samples were also obtained from two AD cases through the UCD Alzheimer’s Disease Center. Tissue specimens were obtained through consented autopsies with their respective institutional review board approvals (UCD and VA West Los Angeles Medical Center). FXTAS patients had all been established through clinical diagnosis based on the presence of intention tremor, cerebellar ataxia, and parkinsonism, and were confirmed to have FXTAS based on the postmortem identification of intranuclear ubiquitin-positive inclusions in brain cells. Control tissue was obtained postmortem from individuals who did not have any significant neurological history, including encephalitis, epilepsy, demyelinating disease, dementia, or concurrent neurodegenerative disease. AD was rated using Consortium to Establish a Registry for Alzheimer’s Disease (CERAD) criteria and Braak stage. All other brain tissues were neuropathologically examined and diagnosed according to clinical symptoms, gross features, and microscopic features. Samples were collected at the time of death and stored at -80 °C until used. Frozen tissue was dissected from the frontal cortex. High-premutation (hpCGG) and wild type (WT) mice with the C57BL/6 background were housed under standard vivarium conditions. All animal use protocols were approved by the Institutional Animal Care and Use Committee at UCD. Cerebral cortex was dissected and flash frozen in liquid nitrogen immediately after mice were sacrificed, then stored at -80 °C until used. Fibroblasts and lymphocytes used were collected and cultured as previously described in Pretto et al. [113]. All sample information is provided in Additional file 1: Tables S1-S3.

### Immunofluorescence

Slides of brain nuclei were prepared using methods described previously [57, 58]. Slides were permeabilized in PBS + 0.5% Triton X-100 for 15 min at room temperature, then blocked in blocking buffer [3% bovine serum albumin (BSA) 0.5% Tween-20, in 1x PBS] for 30 min at room temperature. Primary antibody was diluted to desired concentration in detection buffer (1% BSA, 0.5% Tween-20, in 1x PBS), and slides were incubated in primary antibody for either 1 hr at room temperature or overnight at 4 °C in a humid chamber. After washing in PBS + 0.1% Tween-20, the slides were incubated in secondary antibody for 1 hr at room temperature in a humid chamber and stained with DAPI before coverslips were mounted with either SlowFade Antifade Mountant (for immediate viewing) or Prolong Diamond Antifade Mountant (for longer term viewing or storage). For each immunofluorescence experiment, secondary antibody-only slides and DAPI-only slides were prepared for comparison. Immunofluorescence primary antibodies: rabbit anti-SUMO 2/3 (Abcam ab3742; diluted 1:200), mouse anti-SUMO 2/3 (Abcam ab81371; diluted 1:500), rabbit anti-p62/SQSTM1 (Invitrogen 701510; diluted 1:100), mouse anti-ubiquitin (Bio-rad MCA-1398G; diluted 1:200), rabbit anti-ubiquitin (Abcam 134953; diluted 1:100). Secondary antibodies: goat anti-rabbit Alexa-647 (Abcam ab150083; diluted 1:500), goat anti-mouse Alexa-647 (Abcam ab150115; diluted 1:500), goat anti-mouse Alexa-488 (Abcam ab150113; diluted 1:500), goat anti-rabbit Alexa-488 (Abcam ab150077; diluted 1:500), goat anti-rabbit Alexa-532 (Invitrogen A-11009; diluted 1:500).

### DNA fluorescence in situ hybridization (FISH)

To generate DNA FISH probes, biotin-labeled PCR amplicons corresponding to unique regions spanning and surrounding the *FMR1* gene were generated. Metaphase slides were prepared from primary patient fibroblasts by applying colcemid to fibroblasts at ~70% confluency for 3 hr before cells were collected by trypsinization. Cells were incubated in KCl buffer before fixation using 3:1 methanol acetic acid. 0.3-0.5 μg of probe per slide was applied overnight to cells that were permeabilized successively in several buffers containing detergents, formamide, and ethanol. Bound biotin probe was incubated with Streptavidin bound to Alexa-555 (Invitrogen S32355) at a 1:200 dilution before staining with DAPI and mounting with Prolong Diamond Antifade Mountant.

### Inclusion fractionation

Continuous sucrose gradients were prepared and frozen beforehand using sucrose solutions at concentrations of 2.6, 2.5, 2.4, 2.3, and 2.2 M. In thin-walled polyallomer centrifuge tubes, 6 ml of each solution was added and subsequently submerged in a dry ice ethanol bath, starting with the most concentrated at the bottom and progressing upwards to the least concentrated at the top. Frozen gradients were stored at -20 °C.

Nuclear isolation was performed on frozen human cortical tissue using modified methods from Iwahashi et al. [57] and McEwen and Zigmond [94]. Nuclei were centrifuged and resuspended in 800 μl of BDC + NP-40 [40 mM Tris, 10 mM NaCl, 10 mM CaCl_2_, 5 mM MgCl_2_, 0.1% NP-40, Complete protease inhibitor (Roche), RNase inhibitor (NEB), pH 7.9] and 200 U/ml of DNase I (NEB). Nuclei were Dounce-homogenized with a tight pestle on ice for 30 strokes, after which, the sample was rotated at 37 °C for 6 hr with periodic mixing by pipetting to reduce viscoelasticity. An aliquot was removed at this point to serve as total nuclear protein samples for MS. The remaining sample was centrifuged at 16,000 RCF for 10 min at 4 °C, and the pellet containing FXTAS inclusions was resuspended in BCC (20 mM HEPES, 400 mM NaCl, 1 mM DTT, 1 mM EDTA, 1 mM EGTA, Complete protease inhibitor, pH 7.4) with RNase inhibitor and frozen at -80 °C overnight.

Pre-made sucrose gradients were taken from -20 °C and placed at 4 °C overnight to allow thawing. The frozen, resuspended inclusion samples were thawed on ice, centrifuged at 16,000 RCF for 10 min at 4 °C, then diluted to 10 ml of BCC and carefully pipetted onto the top surface of the sucrose gradient, followed by ultracentrifugation at 100,000 RCF for 6 hr at 4 °C. 2 ml fractions were collected through a small needle puncture at the bottom of each centrifuge tube. Each fraction was diluted with BCC, centrifuged at 3,000 RCF for 10 min at 4 °C, then resuspended in BCC + RNase inhibitor and stored at -80 °C until FACS processing. Slides were made of each fraction to confirm presence of inclusions at fractions corresponding to densities of approximately 1.30 g/ml.

### Western blot

Concentrations of protein lysates were measured using the Pierce BCA or MicroBCA assay kit (Thermo Scientific), and either 10 μg or 20 μg of protein per sample was mixed with 1x Tris buffered saline (TBS) and Laemmli buffer (375 mM Tris-HCl, 6% SDS, 4.8% glycerol, 9% 2-Mercaptoethanol, 0.01% bromophenol blue) to obtain a total volume of 30 μl. Samples were held at 95 °C for 5–10 min and allowed to cool to room temperature before loading onto Criterion TGX 18-well Any kD polyacrylamide gels (Bio-rad) alongside the Chameleon Duo protein ladder (Li-Cor). Samples were run at 20 mA for 20 min until clearly stacked in the gel, then run at 80 mA for 45 min. Gel was then transferred overnight at 4 °C onto a nitrocellulose membrane. The membranes were stained for total protein using Revert Total Protein Stain (Li-Cor) and imaged immediately for total protein on a Li-Cor Odyssey Imager before incubating in blocking buffer (5% BSA in 1x TBS) at room temperature for 1 hr. Primary antibodies were diluted in detection buffer (5% BSA + 0.1% Tween-20 in 1x TBS) and membranes were incubated in primary antibody overnight at 4 °C. The membrane was washed three times in wash buffer (0.1% Tween-20 in 1x TBS) before incubating in secondary antibody diluted in detection buffer at room temperature for 1 hr. Membranes were again washed three times in wash buffer and once in 1x TBS before imaging on a Li-Cor Odyssey Imager. Image processing and densitometry was performed using ImageStudio software. Specific protein signal was normalized both to adjacent background on the image and to total protein signal for each lane. Western blot primary antibodies: rabbit anti-SUMO 2/3 (Abcam ab3742; diluted 1:1,000), rabbit anti-p62/SQSTM1 (Invitrogen 701510; diluted 1:500). Secondary antibody: IRDye 800CW donkey anti-rabbit (Li-Cor; diluted 1:20,000).

### Fluorescence-activated cell sorting (FACS) purification of inclusions

**Fig. 1:**
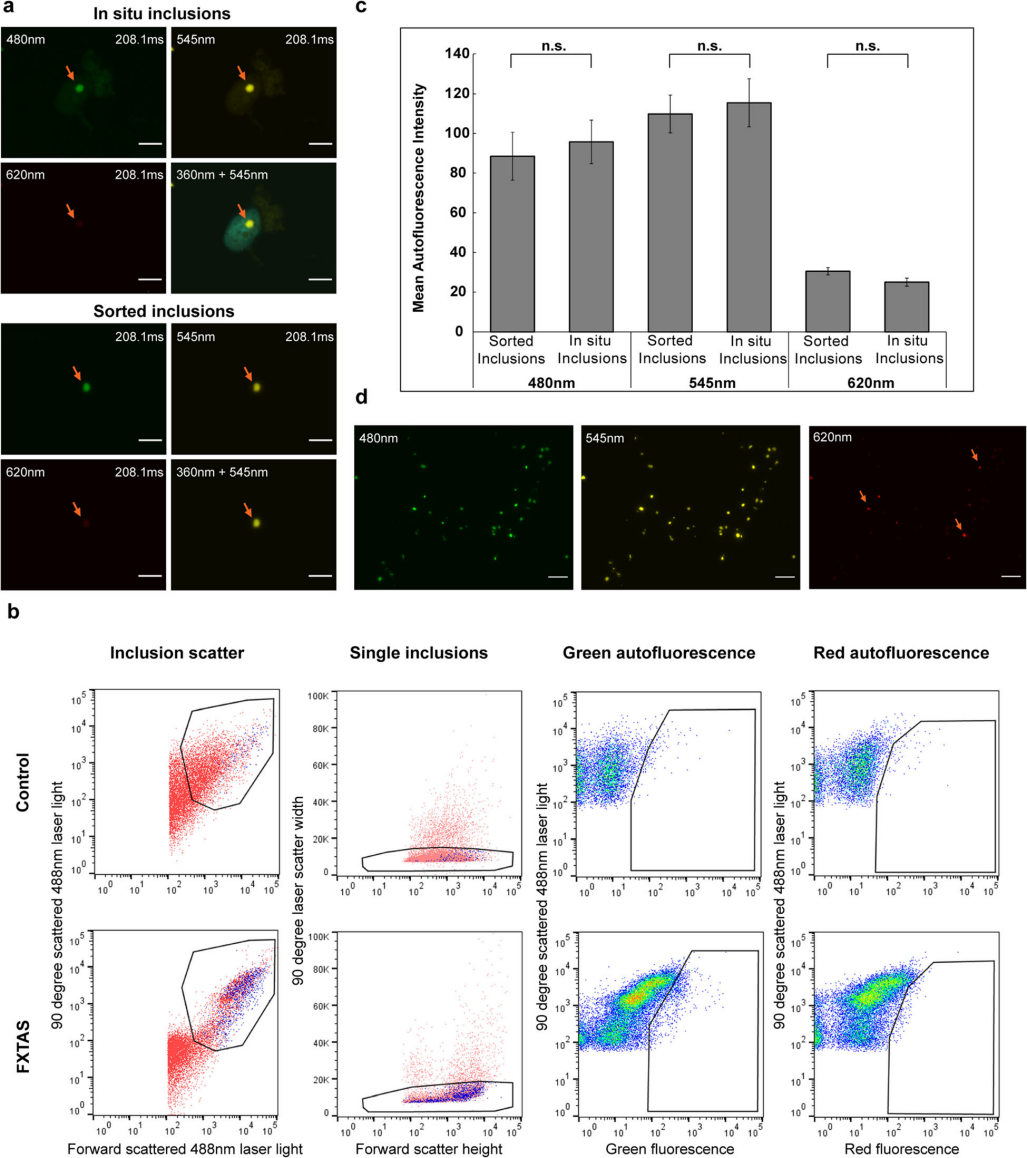
**a,c** Flow-sorted FXTAS inclusions exhibit the same size and autofluorescent properties as *in situ* FXTAS inclusions. FXTAS inclusions exhibit strong autofluorescence at 480 nm and 545 nm, and weak autofluorescence at 360 nm and 620 nm wavelengths. Inclusions sorted by flow cytometry were verified by microscopy to confirm that sorted inclusions exhibit no significant difference from FXTAS inclusions viewed *in situ.* Slides were stained with DAPI only and viewed at 100x. Orange arrows denote inclusions, upper left labels indicate the wavelength used for the image, and upper right labels indicate the exposure level used to take the image. No postprocessing adjustments were made to brightness/contrast. **b** Fractions enriched in inclusions and submitted for FACS contain a population of FXTAS-specific particles identified by size and fluorescence properties. Logarithmic scaling was used on the detectors assigned to laser light scatter measurements (Inclusion scatter), and larger aggregates were removed by plotting the duration of 90° laser light scatter to remove objects with markedly increased laser dwell rates relative to the shorter transit times of single particles (Single inclusions). Sorted particles were identified as a population in FXTAS samples that was absent in control samples which exhibited strong green fluorescence emission and weak red fluorescence emission (gates in Green autofluorescence and Red autofluorescence, respectively). **d** Inclusions sorted sequentially by nuclear isolation, sucrose gradient, and flow cytometry are of high concentration and purity. Sorted inclusion samples viewed at 60x show high purity, with 80-90% of autofluorescent particles displaying autofluorescent properties consistent with FXTAS inclusions. 10-20% of particles (orange arrows) display a high level of autofluorescence in the far-red wavelengths, indicating that a small degree of non-inclusion debris may be present. Scale bars = 5 μm

To sort inclusions for MS, two FXTAS patients (cases B3 and B6 from Table S1) were chosen for analysis alongside one control sample (case B8 from Table S1). Frozen inclusion-enriched fractions of sucrose density gradients from FXTAS nuclei, and equivalent density fractions from unaffected individuals, were thawed and immediately assessed for particle scatter and intrinsic autofluorescence characteristics by flow cytometry using a Beckman Coulter MoFlo Astrios EQ cell-sorting flow cytometer. As observed by fluorescence microscopy (Fig. 1 a), the inclusions present in FXTAS tissue homogenates were small, relatively homogenous in size, and primarily exhibited green autofluorescence (500–565 nm) following 488 nm laser excitation. These green fluorescent particles were not apparent in similarly prepared samples from control tissues. As is standard practice for detection of small subcellular particles [109], we used logarithmic scaling to distinguish inclusions from debris artifacts introduced in the sample buffer. We then removed larger aggregates by plotting the duration of 90° laser light scatter to remove objects with markedly increased laser dwell rates relative to the shorter transit times of single particles. Using these settings, we compared the strength of the autofluorescence signals in FXTAS and control samples across several detectors. We noted that the strongest fluorescence signal was measured in the green detector from 488 nm laser excitation, but this signal was markedly diminished for ≥670 nm wavelengths when subjected to 488, 561, or 640 nm laser excitation. In total, 8.6 million inclusions were sorted from one FXTAS patient sample and 6.5 million inclusions were sorted from a second FXTAS patient sample. Sorted inclusions were centrifuged at 3,000 RCF for 1 hr at 4 °C, and the pellets were pooled for each patient and resuspended in PBS. Aliquots of all samples were taken for the MicroBCA assay. SDS was added to samples to a final concentration of 5% to dissolve insoluble material. Sorted inclusion samples did not contain any visible precipitate after samples were mixed in SDS. MicroBCA assay kits were used according to the manufacturer’s instructions. Sorted inclusion samples yielded comparable total protein concentrations, at 50 μg/ml for the sample containing 6.5 million inclusions, and 68 μg/ml for the sample containing 8.6 million inclusions. Based on these concentrations, inclusions contain approximately 4x10^-6^ μg of protein per inclusion. BCA estimates were used to obtain 25 μg of protein from each sample for MS analysis.

### SUMO 2/3 IP from nuclear lysate

A total of 6 g of frozen frontal cortical tissue, previously powdered under liquid nitrogen, was processed from two FXTAS patients and two age-matched control patients (FXTAS samples B2 and B4 and control samples B7 and B8 from Table S1). Nuclear isolation was performed as described above, except 1 mM EDTA, 1 mM EGTA, and 50 mM N-ethylmaleimide (Millipore Sigma) was added to the buffers. Isolated nuclei were lysed, passed through a 27-gauge needle, heated, then diluted before being centrifuged to remove any remaining insoluble material. Each sample was incubated with SUMO 2/3 antibody for 1 hr at 4 °C with rotation, and Protein G Magnetic Beads (NEB) were incubated with lysate-antibody mixture for 3 hr at 4 °C with rotation. Beads were then placed on a magnetic rack and the supernatant was collected to be run on western blot as the nonbound fraction. Approximately 1/10 of the beads from each IP was separated and eluted using urea elution buffer (7 M urea, 20 mM Tris, pH 7.5, 100 mM NaCl) to be run on western blots as IP elute. The remaining beads were washed three times with 50 mM triethylammonium bicarbonate (TEAB) buffer before on bead digestion.

### Proteomics sample preparation

For proteomic analysis of isolated inclusions and total nuclear protein, sample eluates were dried down in a Centrivap centrifugal vacuum concentrator (Labconco, Kansas City, USA) and reconstituted in 50 μl of SDS solubilization buffer (5% SDS, 50 mM TEAB, 1X PhosSTOP phosphatase and Complete mini protease inhibitor tabs). Samples were clarified by centrifugation at 20,000 RCF for 10 min, supernatants set aside, and the resulting pellet further solubilized with 45 μl 100% formic acid for 1 hr at 37 °C. Solubilized pellets were dried down and recombined with soluble supernatants for analysis. Each volume of normalized sample was enzymatically digested with trypsin using S-Trap micro (Protifi, Huntington, NY) spin columns according to manufacturer instructions with the following modifications: samples were reduced with 20 mM DTT (Millipore Sigma) for 30 min at 56 °C, alkylated with 40 mM IAA (Millipore Sigma) for 30 min at room temperature, and trypsin (Worthington, Lakewood, NJ, USA) was added at a 1:12.5 ratio (enzyme (μg): protein (μg)) and reacted for 2 hr at 47 °C.

For FMRpolyG-GFP heterologous expression samples, harvested cell pellets were lysed directly in 200 μl SDS solubilization buffer with sonication (Qsonica Q125, Newton, CT; 2 rounds of alternating 10 sec on/10 sec off at 20% amplitude). For each sample, protein concentration was determined by BCA assay, volume was normalized to 140 μg of total protein, reduced and alkylated, and enzymatically digested with trypsin using S-Trap mini (Protifi) spin columns according to manufacturer instructions with the following modifications: samples were reduced with 20 mM DTT for 10 min at 50 °C, alkylated with 40 mM IAA for 30 min at room temperature, and two rounds of trypsin were added at a 1:25 ratio (enzyme (μg): protein (μg)) and reacted first for 2 hr at 37 °C followed by a second round overnight at 37 °C.

Sumo-IP samples were on-bead digested with 36 μg trypsin overnight at 37 °C in 50 mM TEAB without reduction or alkylation. Supernatants containing digested peptides were acidified to 1% TFA final concentration. All resulting sample elutes were dried and reconstituted in 2% acetonitrile/0.1% TFA for LC-MS/MS analysis.

### LC-MS/MS and Data Analysis

Digested peptides were analyzed by LC-MS/MS on a Thermo Scientific Q Exactive Plus Orbitrap Mass Spectrometer (Waltham, MA, USA) in conjunction with an EASY-nLC 1200 UHPLC and Proxeon nanospray source operating in positive ionization mode. Peptides were loaded on a 100 μm x 25 mm Magic C18 100Å 5U reverse phase trap before being separated using a 75 μm x 150 mm Magic C18 200Å 3U reverse phase column. Peptides were eluted with an increasing percentage of acetonitrile over the course of a 90- or 120-min gradient with a flow rate of 300 nl/min. An MS survey scan was obtained for the m/z range 350–1600 and acquired with a resolution of 70,000 and a target of 1x10^6^ ions or a maximum injection time of 30 msec. MS/MS spectra were acquired using a top 15 method where the top 15 ions in the MS spectra were subjected to high energy collisional dissociation. MS/MS spectra were acquired with a resolution of 17,500 and a target of 5x10^4^ ions or a maximum injection time of 50 msec. An isolation mass window of 1.6 m/z was used for precursor ion selection, charge states of 2-4 were accepted, and a normalized collision energy of 27% was used for fragmentation. A 20-sec duration was used for dynamic exclusion. For identification and quantitation of data-dependent acquisition data, raw files were searched with Andromeda in MaxQuant version 1.6.1.0 and further processed in Perseus version 1.6.0.2 or Scaffold version 4.8.4.

Additional details for all methods are provided in Additional file 1: Methods.

## Results

### Intrinsic autofluorescence of human FXTAS inclusions facilitates their isolation using preparative flow cytometry

During a series of immunofluorescence studies of FXTAS inclusion-bearing frontal cortex, we observed that the inclusions emit broad-spectrum autofluorescence. Inclusion autofluorescence has previously been reported in neuronal intranuclear inclusion disease (NIID) [70, 110], but has not been reported previously for FXTAS inclusions. Examination of slides generated from multiple FXTAS patients revealed that the inclusions emit the lowest intensity of autofluorescence at 360 nm and 620 nm, with maximal brightness by visual inspection between 480 nm and 545 nm (Fig. 1 a). Stimulated emission depletion (STED) microscopy on a Leica SP8 STED 3x was used to measure the excitation/emission spectra of FXTAS inclusions, and the results corroborated what was seen on visual examination (Additional file 1: Figure S1a), with maximum emission intensity at 550 nm by STED. Autofluorescence has been found in every FXTAS case examined thus far (~10 cases), with spectral properties quantitatively consistent across samples. Additional experiments were performed to ensure that inclusion autofluorescence is not an artifact of fixation, mounting, or staining (unpublished data).

The constancy of FXTAS inclusion autofluorescence has enabled us to isolate relatively pure samples of inclusions using preparative flow cytometry. To minimize contamination due to the known autofluorescence of lipofuscin [27], we isolated nuclei from frozen postmortem cortical tissue by a modification of previous methods [58, 83, 94], followed by sucrose gradient fractionation of disrupted nuclei to obtain a crude inclusion fraction. Our approach, which substantially removes cytoplasmic lipofuscin during the nuclear isolation, largely eliminates non-inclusion autofluorescent cellular material. Ultracentrifugation of nuclear homogenate on a sucrose gradient produces a diffuse band of particles at a density of ~1.30 g/ml, visible in FXTAS samples but not in control samples (Additional file 1: Figure S1b). Visualization of the particles within this band by fluorescence microscopy reveals autofluorescent particles that exhibit the same spectral properties as *in situ* FXTAS inclusions. However, this inclusion-enriched isolate still contains a substantial fraction of non-inclusion particles.

To obtain inclusion samples of greater purity, preparative FACS was used to sort inclusions based on their intrinsic autofluorescence (i.e., without the use of antibodies). The population of particles in the 1.30 g/ml banded material was sorted using size and autofluorescence characteristics (Fig. 1 b). Sorted populations of particles viewed by microscopy exhibit the same spectral properties as *in situ* FXTAS inclusions (Fig. 1 c). FACS-sorted inclusions can be centrifuged and resuspended in a smaller volume to obtain a concentrated sample of relatively pure inclusions (Fig. 1 d). Although most of the particles in these sorted samples exhibit the same spectral properties as inclusions, some contaminants appear too bright at 620 nm to be considered as inclusions and are likely lipofuscin particles that were not completely removed during the nuclear isolation process. Taking these into account, we estimate that FACS sorting inclusion preparations are of 80–90% purity.

### FXTAS inclusions appear to be comprised mainly of protein, with a smaller component of RNA, but do not contain significant amounts of DNA

**Fig. 2:**
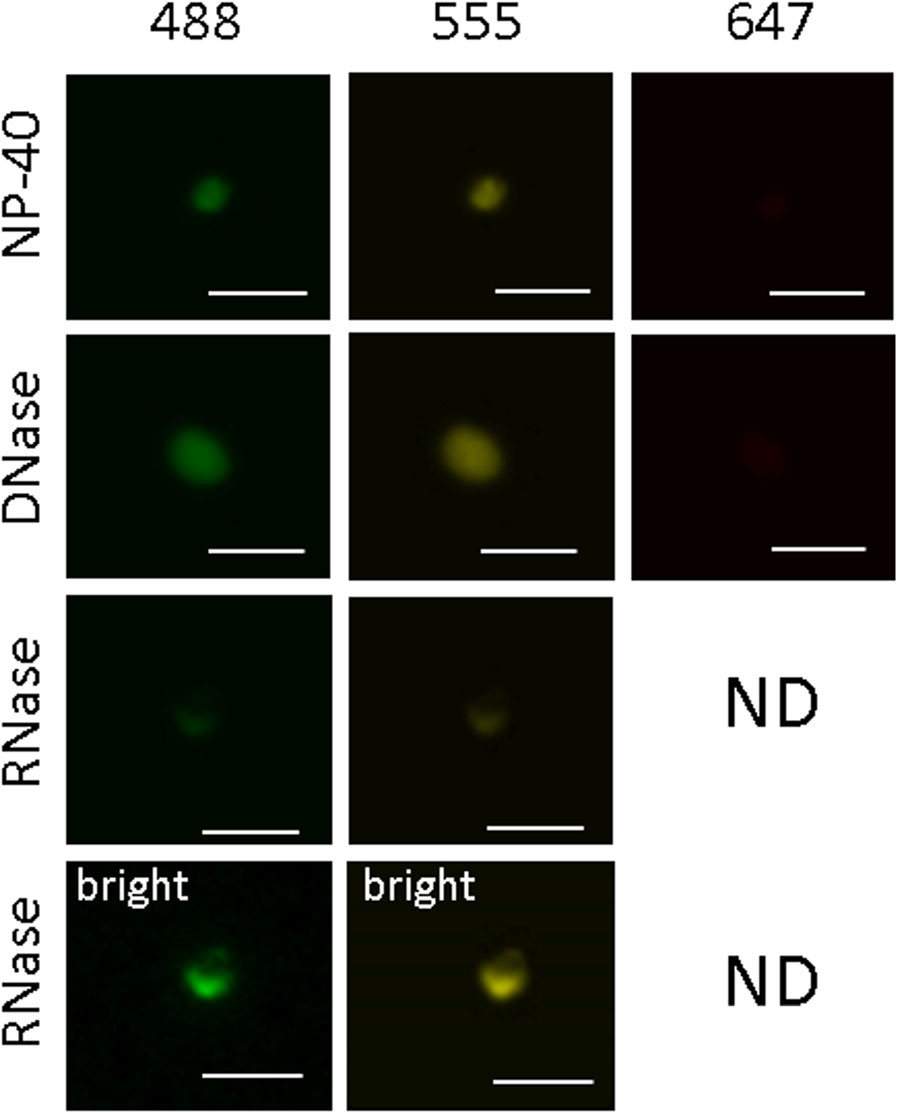
FXTAS inclusions are largely made up of protein and contain a smaller component of RNA, but do not contain significant amounts of DNA. DNase treatment did not affect the quantity, integrity, or level of autofluorescence exhibited by isolated inclusions, detected at 488nm, 555nm; not visualized at 647nm without several fold increase in brightness. RNase treatment did not affect the quantity of inclusions seen, but the autofluorescence levels exhibited by inclusions was decreased. Postprocessing to increase the brightness of RNase treated inclusions revealed that the integrity of the inclusions appeared compromised. Protease treatment completely abolished visualization of inclusions using autofluorescence, and no inclusions were observed. Unless specified (bright), all images were acquired for 208.1 ms

Inclusion-enriched sucrose fractions (1.30 g/ml density) were used to provide a crude estimate of the general molecular composition of inclusions. The inclusions were treated with either DNase I, RNase A/T1, Proteinase K, or no enzyme; each sample was viewed by fluorescence microscopy utilizing autofluorescence (no immunostaining). The no-enzyme and DNase I treatments exhibited wholly intact inclusions, with no diminishment of autofluorescence intensity, inclusion size, or integrity, indicating that DNA does not comprise a significant proportion of the makeup of inclusions (Fig. 2). In comparison, RNase treated inclusions displayed substantial loss of autofluorescence intensity, with many of the inclusions, while still present, appearing to have lost structural integrity. This observation suggests that RNA may play a significant role in the structural makeup of the inclusions, which supports previous evidence that *FMR1* mRNA is present in inclusions [135]. However, preliminary attempts to analyze the RNA complement through RNAseq, although confirming the presence of mRNA, were confounded by extensive degradation of the RNA isolated from the inclusions. Further efforts to determine the composition of the RNA species within the inclusions are ongoing. Finally, Proteinase K treatment alone completely abolished visualization of any inclusions, suggesting that the inclusions are predominantly proteinaceous.

### FXTAS inclusions are primarily heterogeneous protein aggregates enriched for proteins involved in RNA binding and protein turnover and degradation

**Fig. 3:**
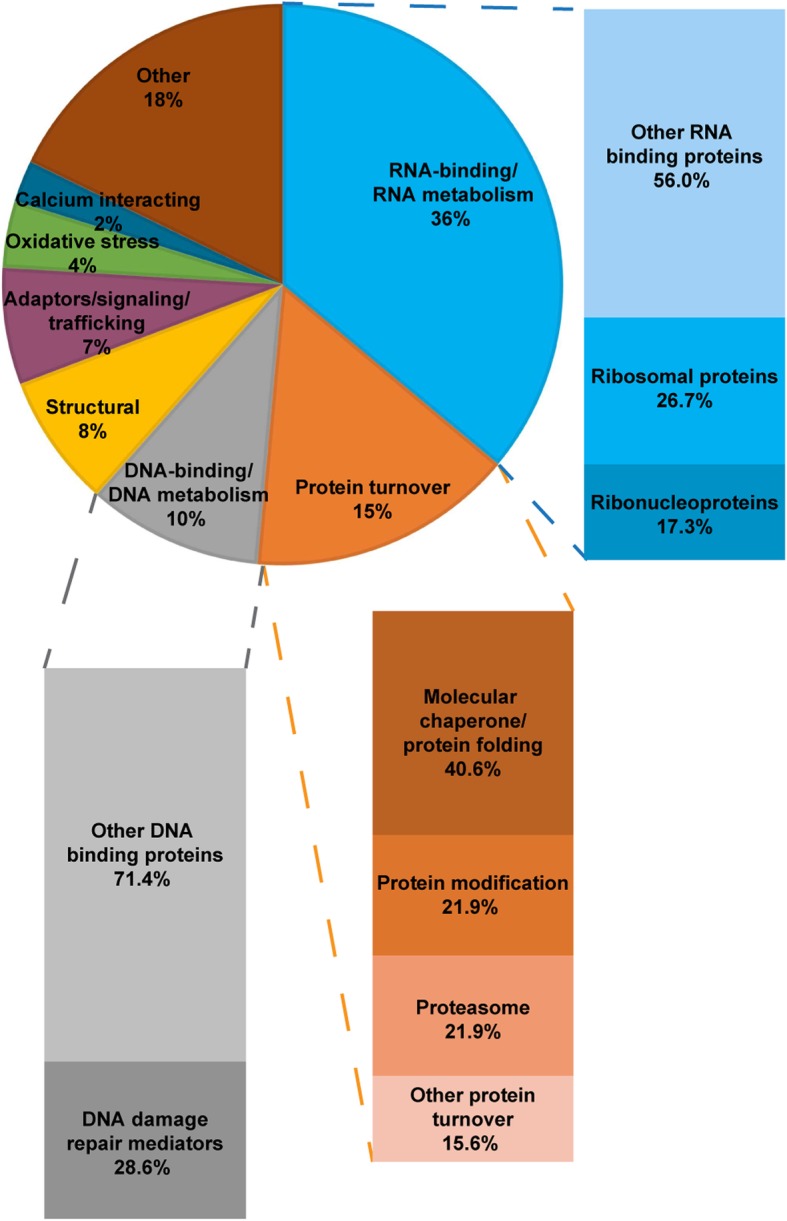
Categorization by function of inclusion-enriched proteins reveals large populations of proteins involved in RNA binding and protein turnover. Proteins that were found present to at least 0.01% in both FXTAS sorted inclusion samples and showed at least 50% enrichment in sorted inclusion samples over accompanying total nuclear samples were identified for categorization. A total of 176 proteins fit these criteria. Proteins were scored by main function(s) as identified by UniProt, and each protein could have multiple scores. Percentages were calculated as number of proteins with that score out of the total number of scores given in the dataset. The “Other” category contains proteins involved in functions including biosynthetic processes, energy metabolism, immunity, and neural development, with no one single category exceeding 3%. A total of 75 proteins were scored as RNA binding proteins and proteins participating in RNA metabolism (transcription, editing, splicing, transport). Many of the higher abundance proteins in this category were hnRNPs. Although there were many ribosomal proteins found in this dataset, almost all of them were found at low abundance (<0.1% of the total protein composition) and were not found to have more total nuclear enrichment in FXTAS nuclei compared to control nuclei. We hypothesize that these proteins represent mainly background through the isolation process and are present more as bystanders rather than main players in inclusion formation. A total of 32 proteins were scored as those participating in protein turnover (protein folding, aggregation, modification, degradation). Within this category, the majority of protein species are those involved in binding and processing proteins destined for recycling or removal. The protein species comprising this category have the highest abundance levels in inclusions. A total of 21 proteins were scored as DNA binding proteins or proteins participating in DNA metabolism (chromatin remodeling, replication, repair). Of these, 6 are players in DNA damage repair, including RAD50, RPA1, and XRCC6

MS-based proteomics was utilized to determine the protein composition of FXTAS inclusions purified from frontal cortex of two FXTAS patients (cases B3 and B6 from Table S1) with high loads of inclusions (~9–14% inclusion-bearing neuronal/astrocytic cells), as previously determined through immunohistochemistry. Patient samples were processed by nuclear isolation/sucrose gradient centrifugation/preparative FACS to obtain sorted inclusion samples. These samples were compared by LC-MS/MS to total nuclear protein from the same samples, as well as to the nuclear isolate from a control non-FXTAS sample (case B8 from Table S1). In general, there was no dominant protein among the inclusion-enriched proteins (see full MS dataset in Additional file 1). Although histones comprised 25–70% of each inclusion protein isolate, none of the histone species was enriched in inclusions over total nuclear protein. The next highest abundance protein, ubiquitin, only comprises ~5% of the inclusion protein complement. To identify proteins likely to be specifically involved in inclusion formation, only those proteins whose molar fractions equaled or exceeded 0.01% of the total inclusion protein content for both sorted inclusion samples and showed at least 1.5-fold enrichment in both sorted samples over their accompanying total nuclear samples were considered for further analysis. One-hundred seventy-six proteins fit these criteria out of a total of ~1,900 proteins identified by MS analysis of the purified inclusions (Additional file 2). The enriched proteins were scored and categorized by function according to UniProt. Three major functional categories stood out among the enriched proteins: RNA binding proteins, proteins involved in protein turnover, and DNA binding proteins (Fig. 3).

Over one-third of the inclusion-enriched proteins are RNA binding proteins. Within this category, 17.3% are ribonucleoproteins. Many of these ribonucleoprotein species (mainly hnRNPs) are present at relatively high abundance within the enriched fraction, half making up over 0.2% in both inclusion samples, and two, hnRNPA1 and hnRNPA3, comprising 1-2% of both inclusion samples. A further 26.7% of the inclusion-enriched RNA binding proteins are ribosomal proteins. However, almost all of these species were present at low abundance (<0.1% of total inclusion protein). They also were not found at higher levels in FXTAS total nuclear samples compared with control total nuclear samples. We also detected approximately forty proteins involved with RNA splicing, the most abundant being U2AF (~0.2%) and SFPQ (~0.5%); both are slightly enriched in the inclusion fraction (by 2.0- and 1.4-fold, respectively). However, there does not appear to be any overall trend for enrichment, with the majority of detected splicing factors being slightly less abundant in the inclusions than in the surrounding nuclear matrix.

A further 15% of inclusion-enriched proteins were scored as involved in protein turnover; that is, proteins that are tasked with binding and processing of other proteins for recycling or removal. Although the protein turnover category contains fewer members compared to the RNA binding category, the protein species comprising this category have the highest abundance levels in inclusions. Within this category, 40% of members were categorized as molecular chaperones, 22% play roles in protein modification, and 22% were members of proteasomal machinery.

The final major functional category, DNA binding proteins, makes up 10% of the population of inclusion-enriched proteins, and 28.6% of these were categorized as DDR mediators. Among these DDR mediators were proteins such as RAD50, RPA1, and XRCC6.

For a more focused analysis of the highest-abundance inclusion-enriched proteins, those proteins which make up at least 0.5% of a sorted inclusion sample and which were enriched by at least 50% were considered further. Only 15 proteins fit these criteria (Table 1), with the most prominent members being ubiquitin and SUMO 2. Other proteins in this list include several hnRNPs and chaperones. Only five proteins were enriched by at least three-fold in both sorted inclusion samples: ubiquitin, SUMO 2, myeloid leukemia factor 2 (MLF2), myelin basic protein (MBP), and p62. Western blot to confirm MS results for p62 was performed on five replicates of control versus FXTAS brain nuclear lysates from control (n=3) and FXTAS (n=4) patients. p62 was seen at 62 kDa and was approximately three-fold more intense in FXTAS samples compared with control samples (Additional file 1: Figure S2a), and immunofluorescence on FXTAS brain smears confirmed the presence of p62 in nuclear inclusions (Additional file 1: Figure S2b).

**Table 1:** Higher abundance proteins enriched at least 1.5x in FXTAS inclusions include protein modifiers, RNA binding proteins, and chaperone proteins

	FXTAS A Inclusions		FXTAS B Inclusions	
Proteins	Over FXTAS A nuclear	Over control nuclear	Over FXTAS B nuclear	Over control nuclear
Small ubiquitin-related modifier 2 (SUMO2)	**5.5 (0.60/0.11)**	**60.0 (0.60/0.01)**	**9.1 (4.37/0.48)**	**437.0 (4.37/0.01)**
p62/ SQSTM1	**8.0 (0.08/0.01)**	**40.0 (0.08/0.002)**	**30.0 (0.60/0.02)**	**300.0 (0.60/0.002)**
Myeloid leukemia factor 2 (MLF2)	**9.3 (0.28/0.03)**	**93.3 (0.28/0.003)**	**27.3 (0.82/0.03)**	**273.3 (0.82/0.003)**
Ubiquitin (RS27A)	**3.6 (0.65/0.18)**	**5.9 (0.65/0.11)**	**6.7 (5.17/0.77)**	**47.0 (5.17/0.11)**
Myelin basic protein (MBP)	**3.5 (0.46/0.13)**	**1.2 (0.46/0.38)**	**15.2 (0.76/0.05)**	**2.0 (0.76/0.38)**
Heat shock protein HSP 90-alpha (HSP90AA1)	1.6 (0.08/0.05)	4.0 (0.08/0.02)	1.7 (0.67/0.39)	33.5 (0.67/0.02)
Heterogeneous nuclear ribonucleoprotein L (HNRNPL)	1.9 (0.13/0.07)	6.5 (0.13/0.02)	1.6 (0.52/0.32)	26.0 (0.52/0.02)
Heterogeneous nuclear ribonucleoprotein A1 (HNRNPA1)	1.7 (1.12/0.65)	2.7 (1.12/0.41)	1.6 (2.16/1.32)	5.3 (2.16/0.41)
Heterogeneous nuclear ribonucleoprotein A3 (HNRNPA3)	1.8 (0.95/0.54)	2.6 (0.95/0.36)	1.6 (1.59/0.97)	4.4 (1.59/0.36)
Heterogeneous nuclear ribonucleoproteins C1/C2 (HNRNPC)	1.9 (0.28/0.15)	1.3 (0.28/0.22)	2.0 (0.59/0.30)	2.7 (0.59/0.22)
Beta-actin (ACTB)	2.2 (0.75/0.34)	1.2 (0.75/0.61)	1.8 (1.33/0.75)	2.2 (1.33/0.61)
Alpha-crystallin B chain (CRYAB)	1.9 (1.29/0.68)	1.1 (1.29/1.22)	1.7 (2.66/1.53)	2.2 (2.66/1.22)
Tubulin alpha-1B chain (TBA1B)	1.6 (1.01/0.63)	1.0 (1.01/0.98)	1.6 (0.81/0.50)	0.8 (0.81/0.98)
Tubulin beta-2A chain (TBB2A)	1.7 (0.64/0.37)	0.9 (0.64/0.71)	1.5 (0.58/0.39)	0.8 (0.58/0.71)
Calcium/calmodulin-dependent protein kinase type II subunit alpha (CAMK2A)	1.7 (0.43/0.25)	0.7 (0.43/0.61)	1.6 (0.59/0.38)	1.0 (0.59/0.61)

Only five of these proteins (bolded) were enriched at least 3-fold in FXTAS inclusions over accompanying FXTAS total nuclear proteins, and only SUMO 2/3, MLF2, and p62 were also found to be enriched at least 3-fold in FXTAS total nuclear samples over control total nuclear sample

Fold change values for each of the FXTAS inclusion samples over their accompanying FXTAS nuclear samples and the control nuclear sample are shown, with the abundance values as a percentage of the total identified protein molar composition for each sample displayed in parentheses. Proteins identified were present in at least one FXTAS sorted inclusion sample to at least 0.5% and showed at least 50% enrichment in both FXTAS sorted inclusion samples over corresponding FXTAS nuclear samples. Only five of these proteins (bolded) were enriched at least 3-fold in FXTAS inclusions over accompanying FXTAS total nuclear proteins, and only SUMO 2/3, MLF2, and p62 were also found to be enriched at least 3-fold in FXTAS total nuclear samples over control total nuclear sample. Numeric values presented outside parentheses are fold change of inclusion samples over nuclear samples. Values inside parentheses show the abundance values as a percentage of the total protein composition identified for inclusion samples over nuclear samples.

### SUMO 2/3 conjugates are present in FXTAS brain nuclei at levels exceeding ten-fold over the levels found in brain nuclei from controls

**Fig. 4:**
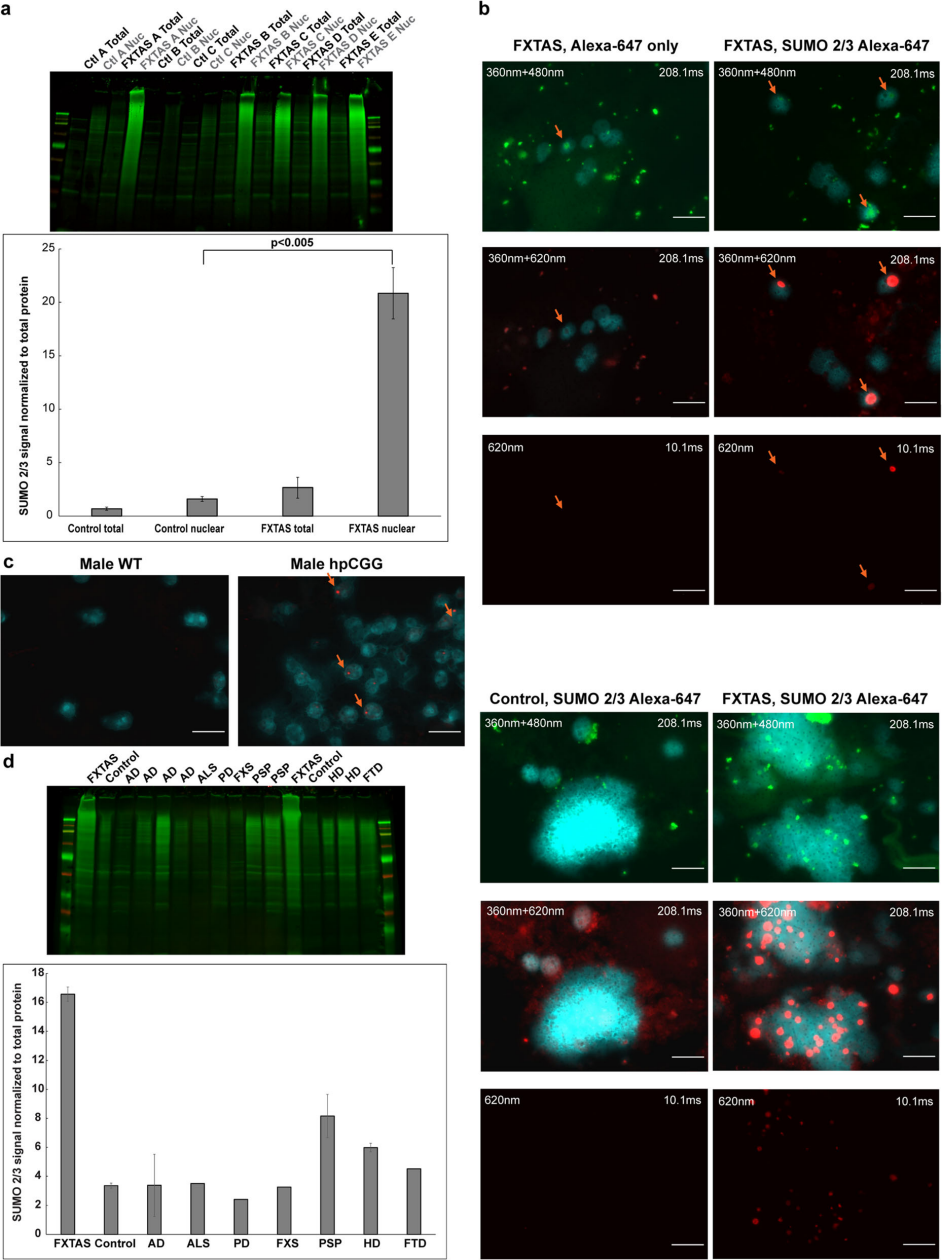
**a** FXTAS nuclear protein samples exhibit over ten times higher SUMO 2/3 protein levels compared to control nuclear protein samples. FXTAS nuclear protein samples exhibit a vibrant SUMO 2/3 smear indicating over 10 times higher levels of conjugated SUMO 2/3 over control nuclear protein samples after normalizing to total protein levels (unpaired student’s t-test (single group) with p-value <0.005). Whole tissue sample signals were extremely faint by comparison. No significant difference was seen between control and FXTAS samples for unconjugated SUMO 2/3. **b** SUMO 2/3 immunofluorescence reveals that SUMO 2/3 is present mainly as large, concentrated intranuclear aggregates in FXTAS brain cells, and FXTAS inclusions always colocalize with SUMO 2/3 aggregation. SUMO 2/3 forms large, intranuclear aggregates in FXTAS brain cells that colocalize with FXTAS inclusions. Immunofluorescence was performed using an Alexa 647 secondary antibody to minimize the effect of inclusion autofluorescence. 360nm+480nm images show inclusion autofluorescence. In FXTAS samples, SUMO 2/3 aggregates at 620nm are so vibrant that the signal intensity at 208.1ms is distorted, and the signal is bright even at an exposure of 10.1ms. No post-processing was done to alter brightness/contrast levels. Orange arrows denote FXTAS inclusions. When looking at clusters of nuclei (bottom panels), control brain cells show diffuse SUMO 2/3 staining throughout nuclei, but no aggregates, whereas FXTAS brain cells show large, circular aggregates in a large proportion of cells. **c** SUMO 2/3 aggregates are present in mouse high premutation CGG brain nuclei but are not present in wildtype brain nuclei. Intranuclear SUMO 2/3 aggregates are also seen in 11-month-old premutation CGG male mouse brain, but not in age-matched male wildtype mice. Aggregates were smaller and fewer than those seen in human FXTAS brain. Immunofluorescence was performed using an Alexa-647 secondary antibody. No postprocessing was done to alter brightness/contrast levels. Orange arrows denote SUMO 2/3 aggregates. **d** The SUMO 2/3 smears found in FXTAS nuclear protein samples are not present to the same degree in other neurodegenerative brain samples investigated. Western blot analysis was performed on nuclear lysates from human frontal cortex tissue obtained from a variety of neurodegenerative disorders. Due to the lack of availability of sufficient material, replicates were not possible for several samples. Where replicates were possible, error bars are shown. Scale bars = 10 μm

To confirm elevation of SUMO 2 in FXTAS brain nuclei, western blot analysis was performed using a SUMO 2/3 antibody on protein lysates from whole brain tissue and nuclear brain protein lysates from FXTAS (n=5) patients and controls (n=3) (Fig. 4 a, Additional file 1: Figure S3). Although MS analysis can distinguish SUMO 2 from SUMO 3, most antibodies recognize both species due to their high level of sequence similarity. Each FXTAS nuclear sample exhibited an intense SUMO 2/3 smear extending from the well down to around 25–30 kb, indicating an unusually large amount of conjugated SUMO 2/3, especially at higher molecular weights. This smear was not apparent in whole brain tissue lysates, indicating that the SUMO conjugates are present primarily or exclusively in the nuclear compartment, with the conjugates being diluted in whole tissue. Quantification revealed an over ten-fold elevation of conjugated SUMO 2/3 in FXTAS nuclei compared to control nuclei. Interestingly, no significant differences were seen between FXTAS and control for the SUMO 1 isoform (Additional file 1: Figure S4).

Immunofluorescence of control and FXTAS brain nuclei was used to localize the elevated SUMO 2/3. Staining was performed in the far-red spectral region (620 nm) where autofluorescence is minimal; secondary antibody-only controls are shown for comparison. SUMO 2/3 staining produced an intensely bright signal within inclusions (Fig. 4 b, top panels). All inclusions that were identified by autofluorescence at 480 nm stained intensely positive for SUMO 2/3 at 620 nm. In addition, bright SUMO 2/3 aggregates found at 620 nm could be used to identify smaller inclusion-like aggregates that were not immediately apparent at 480 nm or 545 nm. This suggests that SUMO 2/3 accumulation may be an early and major participant in inclusion formation. Outside of the SUMO 2/3 aggregates, there did not appear to be any generalized increase in SUMO signal in the rest of the nucleus compared to control. The quantity of SUMO-staining aggregates became apparent when looking at incompletely homogenized clumps of cells in control versus FXTAS brain, where SUMO staining only lightly and diffusely stained the cells in control, whereas many SUMO 2/3 aggregates were present in FXTAS (Fig. 4 b, bottom panels).

The same analysis of SUMO 2/3 immunofluorescence was performed on 11-month-old, high premutation CGG (hpCGG; ~170 CGG repeats), and wildtype (WT) mouse brain tissue to determine whether SUMO 2/3 aggregation also occurs in an *FMR1* premutation mouse model [10, 142]. Many SUMO 2/3 aggregates were apparent in both male and female homozygous hpCGG mouse brain but were not present in age-matched WT mice (Fig. 4 c, Additional file 1: Figure S5a). However, the number and size of the aggregates were smaller than those present in human brain. Western blot analysis of hpCGG mouse brain nuclear lysates did not exhibit the same prominent SUMO 2/3 smears present in human FXTAS brain, perhaps because the premutation mice were still in the premutation phase (only 11 mo old) whereas FXTAS patient postmortem brains were in the later, neurodegenerative phase (>65 yr old) (Additional file 1: Figure S5b). The same hpCGG slides were also examined for inclusion autofluorescence, which was not detected. Finally, very few ubiquitin-staining inclusions were found on the entire slide used to detect SUMO 2/3 immunofluorescence, consistent with the ~1.6% of cells with inclusions in frontal cortex for these premutation mice at 11 months of age [142], which was a much lower percentage of inclusion-bearing cells compared to SUMO 2/3 aggregate-bearing cells. This finding indicates that SUMO 2/3 aggregation precedes the formation of canonical ubiquitin-staining FXTAS inclusions.

### The levels of SUMO 2/3 in nuclei from FXTAS brains exceed the levels found in several other common neurodegenerative disorders

To determine whether the elevated SUMO 2/3 in FXTAS is specific to this disorder or is representative of a generalized neurodegenerative phenomenon, a variety of other neurodegenerative brain samples were collected for nuclear isolation followed by western blot (Fig. 4 d, Additional file 1: Figure S6). No other disease consistently exhibited elevated SUMO 2/3 immunoreactivity to the same degree as FXTAS, but several other brain samples from patients exhibiting neurodegenerative disease did exhibit higher than normal levels of SUMO 2/3, including FTD, HD, and PSP. Although one patient diagnosed with AD did show an abnormally high SUMO 2/3 signal, this was not consistently recapitulated in other AD samples. Brains from patients diagnosed with PD, ALS, and FXS exhibited SUMO 2/3 levels similar to that of control brains. These results are preliminary, as more replicates were not possible due to insufficient sample quantity, but they indicate that the consistently high level of SUMO 2/3 seen in FXTAS samples is unique to this disorder; although, SUMO 2/3 may play a role in other inclusion and/or aggregate-bearing neurodegenerative diseases. Samples tested in the current study were nuclear lysates, so diseases bearing cytoplasmic-only inclusions, such as PD and ALS, may have aggregates that contain SUMO 2/3 in their cytoplasmic compartments that would not be captured here. The role of SUMO 2/3 in other neurodegenerative disorders warrants further study.

### SUMO 2/3 immunoprecipitation proteomics reveal that SUMO 2/3 is highly conjugated to proteins involved with protein turnover, DNA damage/repair, and protection against oxidative stress/damage in FXTAS

To further assess the targets of SUMO 2/3 conjugation in FXTAS patients, we performed IP with a SUMO 2/3 antibody followed by LC-MS/MS to determine what population of proteins are modified by SUMO 2/3 in FXTAS patients compared to controls. IP methods were adapted from several sources [50, 52, 145, 155]. In brief, nuclei were isolated from frontal cortex tissue of two FXTAS cases and two controls. Nuclei were lysed and heated in an SDS/Triton/deoxycholate buffer, then diluted to minimize detergent interference for IP. Pulldown was performed using a SUMO 2/3 mouse IgG antibody and anti-mouse IgG magnetic beads. Western blot confirmed that the pulldown successfully captured the SUMO 2/3 smear (Additional file 1: Figure S7). LC-MS/MS of the IP eluted proteins confirmed the high levels of SUMO 2/3 pulldown in the FXTAS samples (Table 2). As with the MS analysis of the FXTAS inclusions, no one protein dominated in the IP, indicating that the elevated conjugated SUMO 2/3 present in FXTAS is not due to its conjugation with one particular protein.

**Table 2:** Among higher abundance proteins that were mildly enriched in FXTAS samples were other protein turnover agents and proteins involved in non-homologous end joining (NHEJ)

Proteins	Averaged FXTAS over Control
p62/SQSTM1	**237.5 (0.19/0.0008)**
Small ubiquitin-related modifier 2 (SUMO2)	**11.4 (9.88/0.87)**
Small ubiquitin-related modifier 3 (SUMO3)	**12.9 (0.09/0.007)**
Small ubiquitin-related modifier 1 (SUMO1)	**7.2 (0.43/0.06)**
Ubiquitin (RS27A)	**3.9 (0.51/0.13)**
Carbonyl reductase [NADPH] 1 (CBR1)	20.5 (0.41/0.02)
14-3-3 protein theta (1433T)	15.0 (0.12/0.008)
Gelsolin (GSN)	11.0 (0.11/0.01)
Peptidyl-prolyl cis-trans isomerase A (PPIA)	5.4 (0.54/0.10)
Triosephosphate isomerase (TPIS)	3.5 (0.07/0.02)
X-ray repair cross-complementing protein 5 (XRCC5)	3.3 (0.20/0.06)
Creatine kinase B-type (KCRB)	3.2 (0.51/0.16)
Poly [ADP-ribose] polymerase 1 (PARP1)	2.7 (0.38/0.14)
Splicing factor U2AF 35 kDa subunit-like protein (U2AF5)	2.6 (0.13/0.05)
Isocitrate dehydrogenase [NADP] (IDHP)	2.3 (0.09/0.04)
X-ray repair cross-complementing protein 6 (XRCC6)	2.0 (0.34/0.17)
Centromere protein V (CENPV)	1.7 (0.38/0.22)
ATP-dependent RNA helicase (DDX1)	1.4 (0.10/0.07)
Profilin-1 (PROF1)	1.3 (0.08/0.06)
Alpha-crystallin B chain (CRYAB)	1.3 (2.11/1.63)

Excluding SUMO 2, proteins that showed at least 3-fold enrichment in both FXTAS samples over age matched control samples are bolded

Fold change values for the averaged FXTAS IP samples over the averaged age-matched control IP samples are shown. Proteins that were present in at least one FXTAS sample to at least 0.1% and showed at least 20% enrichment in both FXTAS samples over age matched control samples are displayed here. The high level of enrichment in SUMO 2 signal present in FXTAS samples confirms the success of the IP. Excluding SUMO 2, proteins that showed at least 3-fold enrichment in both FXTAS samples over age matched control samples are bolded. Numeric values presented outside parentheses are fold change of averaged FXTAS samples over averaged control samples. Values inside parentheses show the averaged abundance values as a percentage of the total protein composition identified for FXTAS samples over control samples.

Among the more abundant SUMO 2/3 targets identified by IP were proteins with chaperone/protein-folding functions, including αβ-crystallin (CRYAB) and peptidyl-prolyl cis-trans isomerase A (PPIA) [13, 48]; conjugating proteins known to be involved in protein turnover, including ubiquitin, SUMO 1, and SUMO 2/3 [79, 96, 102]; and prominently, the ubiquitin-binding shuttling protein p62 [26, 107, 143]. We also detected proteins known to be involved in non-homologous end joining (NHEJ), including poly [ADP-ribose] polymerase 1 (PARP1), X-ray repair cross-complementing protein 6 (XRCC6/Ku70), and X-ray repair cross-complementing protein 5 (XRCC5/Ku80) [22, 146]. Several proteins involved in cellular protection against oxidative stress, such as carbonyl reductase 1 (CBR1) [67, 121, 151] and mitochondrial isocitrate dehydrogenase (IDH2) [47, 61, 69], were also identified. Other than SUMO 2, only 4 proteins were enriched at least 3-fold in FXTAS IPs over control IPs: SUMO 1, SUMO 3, Ubiquitin, and p62.

### FMRpolyG is a minor component in FXTAS inclusions and IP conjugates

**Fig. 5:**
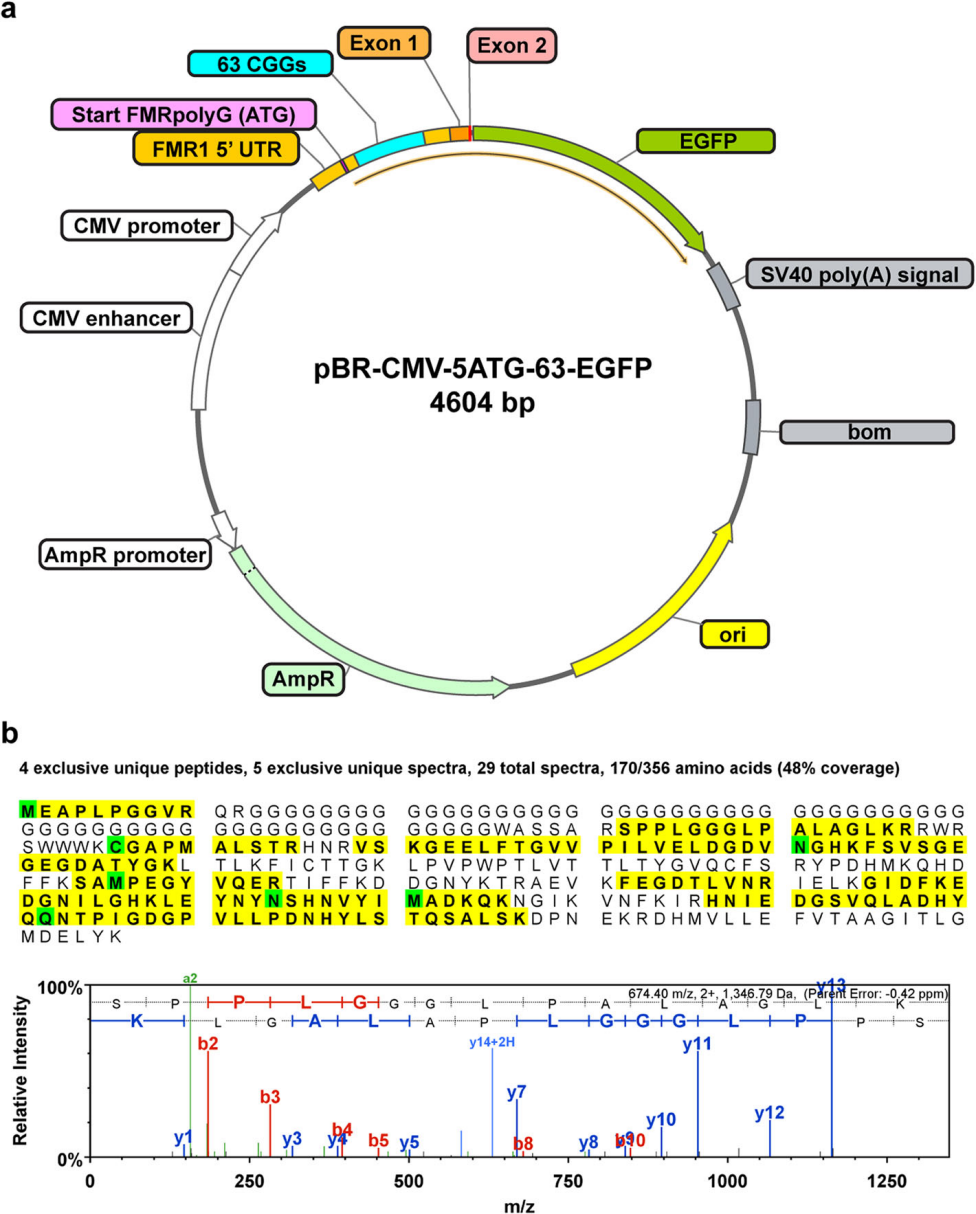
**a** Vector pBR-CMV-5ATG-63-EGFP produces FMRpolyG containing 63 CGG repeats fused to EGFP under a strong CMV enhancer and promoter. As a positive control for FMRpolyG detection by LC-MS/MS, an expression plasmid was generated with a CMV promoter driven FMRpolyG-GFP fusion construct. The human *FMR1* 5′ untranslated region (**UTR**) and Exon 1 sequence, from Transcriptional Start Site I (**TSS-I**) to the first three base pairs of Exon 2, were placed upstream and in frame with EGFP to create a stable fusion protein. The translational start site of FMRpolyG was modified from the native ACG to the canonical ATG to further drive FMRpolyG expression. **b** LC-MS/MS analysis of FMRpolyG-GFP expressed in an SK-N-MC heterologous cell expression system identifies multiple proteotypic peptides. Transiently transfected SK-N-MC cells containing the pBR-CMV-5ATG-63-EGFP plasmid were SDS solubilized and whole cell protein lysates were trypsin digested and analyzed by LC-MS/MS. GFP signal was observed in FMRpolyG and GFP expressing cells, but not null transfection controls, indicating successful transfection of the constructs. Peptide sequence coverage of FMRpolyG-GFP (48%) is indicated in yellow with modified residues in green. LC-MS/MS identified three unique FMRpolyG-GFP peptides present only in FMRpolyG-GFP expressing cells that map to native FMRpolyG protein. A representative mass spectrum of the FMRpolyG specific peptide SPPLGGGLPALAGLK is provided with y/b ions labeled

It has been posited that FXTAS inclusions are largely made up of aggregates of FMRpolyG and interacting proteins [19, 125, 137]. However, endogenous FMRpolyG has never been identified in FXTAS patient samples through direct protein sequencing. Importantly, we have identified FMRpolyG in both sorted inclusion samples and FXTAS SUMO IP samples (Table 3). However, FMRpolyG constituted an extremely minor component, at 0.003% of the total protein molar complement in one inclusion sample and 0.04% in the other sample (~30–400 ppm molar abundance). Importantly, neither of the FXTAS nuclear lysate samples nor control brain contained detectable levels of FMRpolyG. Thus, FMRpolyG can only be detected with significant enrichment. Additionally, two proteins, LAP2β and TRA2A, which have been proposed to co-aggregate with FMRpolyG within inclusions [19, 125], were also found at very low levels. LAP2β was detected at 0.02% and 0.04% in inclusions while TRA2A was detected at 0.0001% in the inclusion samples. As a positive control for detection of FMRpolyG, we expressed an FMRpolyG GFP fusion construct in SK-N-MC neuroblastoma cells (Fig. 5 a). We identified three proteotypic peptides mapping to FMRpolyG-GFP that were not detected in null or GFP transfected cells (Fig. 5 b). Finally, FMRpolyG was also detected in both FXTAS IP samples, but was again at a low abundance (0.01%) and detected through just one hit of one tryptic peptide in each sample (Table 3).

**Table 3:** Identified FMRpolyG proteotypic peptides

Sequence	Modifications	Observed m/z	Charge	Theoretical Mass (Da)	Spectral Counts (MS/MS)		
					FMRpolyG-GFP (recombinant)	Native FXTAS Inclusions	Native FXTAS SUMO-IP
MEAPLPGGVR	Acetyl (+42)	534.78	2	1068.54	1	-	-
	Acetyl (+42) Oxidation (+16)	542.78	2	1084.54	-	1	-
EAPLPGGVR		448.25	2	895.50	-	1	-
SPPLGGGLPALAGLK		674.40	2	1347.80	3	1	2
SPPLGGGLPALAGLKR		501.97	3	1503.90	1	-	-
CGAPMALSTR	Carbamidomethyl (+57)	532.25	2	1063.50	1	-	-
	Carbamidomethyl (+57) Ammonia-loss (-17)	523.74	2	1046.50	1	-	-

### FXTAS inclusions do not co-localize with the *FMR1* gene

**Fig. 6:**
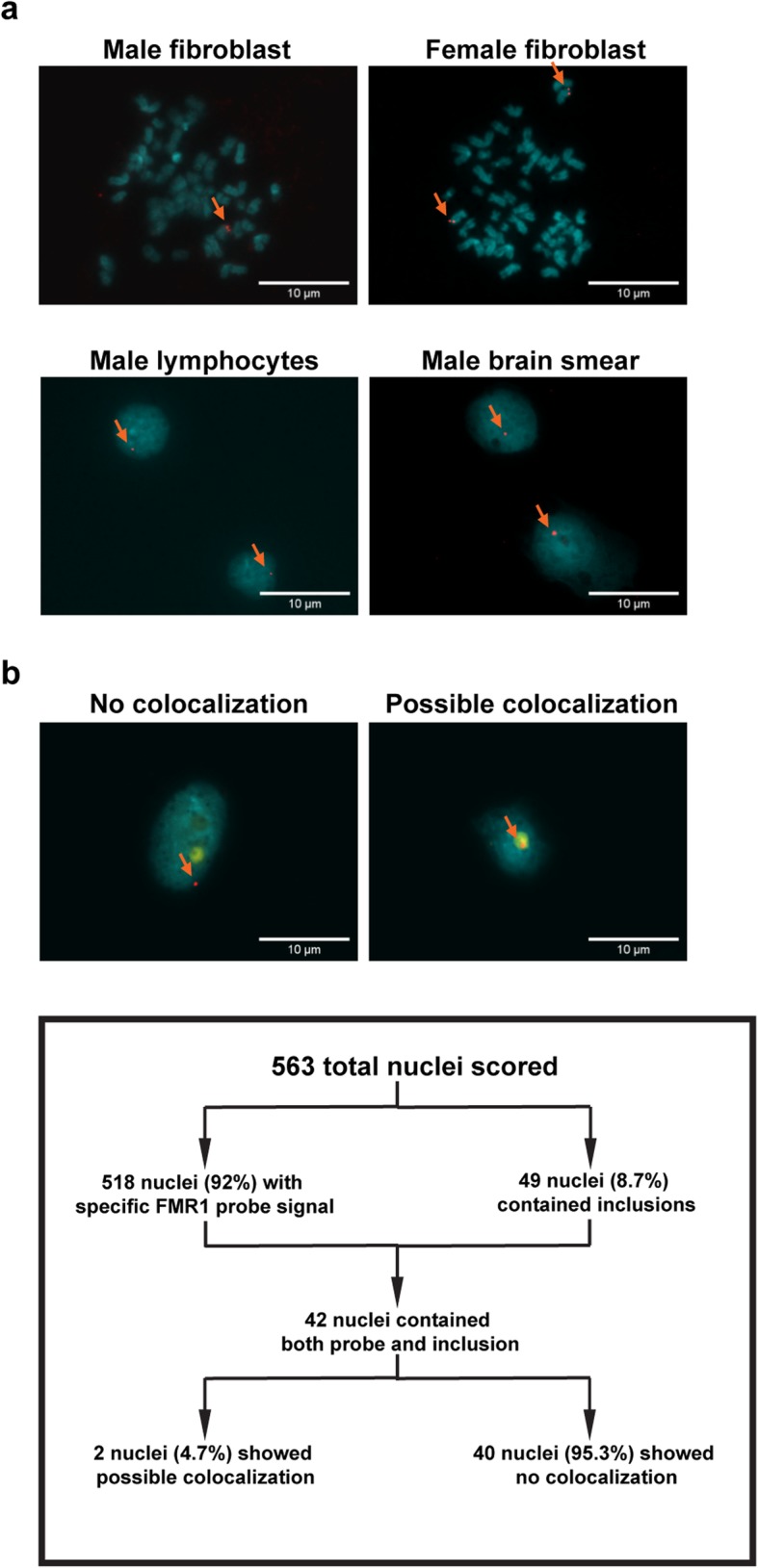
**a** DNA FISH probe generated for the *FMR1* locus successfully tags the DNA locus in fibroblasts, lymphocytes, and brain nuclei. DNA FISH probe generated using PCR amplification to tag the *FMR1* locus cleanly tags the *FMR1* locus at high efficiency in all cell types examined. Metaphase plates generated using control patient-derived fibroblasts exhibit expected tagging patterns, with male samples exhibiting one tag located towards the end of the long arm of the X chromosome, and with female samples exhibiting two such tags. Scoring of over 1100 fibroblast cells in a grid-like fashion showed 97% of cells exhibited sensitive and specific binding. Patient-derived lymphocytes exhibited similar binding and efficiency. Brain nuclei exhibited slightly lowered binding efficiency at 92%, possibly due to degradation of cells due to *pre* and postmortem conditions. **b** The *FMR1* locus does not preferentially co-localize with inclusions. Out of 563 FXTAS nuclei scored, 518 of the nuclei displayed sensitive and specific probe binding, representing an approximate FISH probe efficiency of 92% in brain tissue. Forty-five nuclei had either no probe signal or multiple probe signals. Forty-nine of the nuclei scored positive for inclusions, representing about 8.7% of the total nuclei present. Forty-two of inclusion-bearing nuclei also showed sensitive and specific probe binding. Forty of these nuclei showed negative colocalization between the *FMR1* probe and inclusion, while 2 nuclei showed possible colocalization between the *FMR1* probe and the inclusion. These data indicate that the *FMR1* gene does not co-localize with FXTAS inclusions

The observation that FXTAS inclusions are exclusively solitary and intranuclear has led to the hypothesis that inclusion formation may occur at the active *FMR1* locus, perhaps forming due to the accumulation of DDR proteins around the damage-prone expanded repeat [32, 42, 43]. To test this, a DNA fluorescence *in situ* hybridization (**FISH**) composite probe set, comprised of 119 individual DNA oligonucleotides, was generated to target the human *FMR1* locus. The FISH probe was generated using a modified PCR protocol to avoid the repeat-rich regions surrounding and including the *FMR1* locus. Probe specificity and efficiency was tested on a variety of human cells, including metaphase and interphase fibroblasts cultured from skin biopsies, lymphocytes, and brain smears made from fresh frozen human frontal cortex tissue (Fig. 6 a). In fibroblasts, over one thousand nuclei from both female and male samples were scored for probe binding, and 97% of nuclei contained clear and specific FISH probe signal. In human brain nuclear smears, over 500 nuclei from one FXTAS patient were scored for FISH probe binding, inclusion presence, and localization of the *FMR1* locus relative to inclusions. 92% of the scored nuclei contained clear and specific probe signal, and 8.7% of the scored nuclei contained nuclear inclusions (Fig. 6 b). Previous reports of inclusion load in FXTAS frontal cortical tissue range from 2–20% of neurons and astrocytes [38], and our quantification of inclusion load based on autofluorescence falls within this range. Of the inclusion- and probe-bearing nuclei, 95.2% of them clearly showed no-colocalization between the inclusion and the *FMR1* locus, and the remaining 4.8% showed possible co-localization (Fig. 6 b). These data do not support the hypothesis that the *FMR1* locus consistently co-localizes with FXTAS inclusions.

## Discussion

A principal outcome of the current study is that the protein complement of the FXTAS intranuclear inclusions is not dominated by a single, enriched protein. Rather, the inclusions comprise a large number of proteins, each present at only a few percent or less of the total proteins identified. The current observations both validate and expand upon an earlier, more limited study of FXTAS inclusions [58]; with a number of proteins identified previously now quantified (see: Additional file 1) as well as many additional protein components identified (e.g. SUMO 2/3, p62). Moreover, the current study has followed up the finding of SUMO2/3 enrichment by using immunoprecipitation to identify proteins to which these modifiers are ligated.

The strong enrichment for proteins involved in protein turnover as well as the overall heterogeneous population indicates that the inclusions are principally repositories of proteins destined for removal. There are several possibilities for why these proteins are aggregating. There is good evidence for the presence of RNA in the inclusions, and particularly *FMR1* mRNA [135], which may serve as driver for aggregation. In support of the involvement of RNA, RNase treatment does affect the integrity of the inclusions (Fig. 2), and 36% of inclusion proteins are RNA binding proteins, which further supports the presence of RNA (Fig. 4). Although RNA sequencing of FXTAS inclusions would be an excellent way to ascertain what RNA species might be playing roles in inclusion formation, efforts to characterize the RNA within the inclusions have not been successful thus far due to extensive degradation of the RNA isolates. This could be due to either the strategy used in this study to isolate inclusions or due to intrinsically degraded RNAs within the inclusions.

In addition, SUMO 2 may play a role in aggregation. SUMO proteins have been well-studied in the context of neurodegenerative disease for the diverse roles that they can play as protein modifiers [4]. Although SUMO 2 was found to co-aggregate with polyQ-ATXN7, and mouse models showed accumulation of SUMO 2 in SCA7 patient brain [90], SUMO 2 had not previously been reported as a major inclusion protein. The intensity of the SUMO 2 signal in FXTAS compared to other neurodegenerative diseases suggests something specific in FXTAS which is not present or not as apparent in other inclusion disorders. Unlike the current study, almost all studies reporting a role for SUMO in other neurodegenerative diseases have found roles for SUMO 1 rather than SUMO 2/3 [29, 85, 132, 152]. SUMO 1 is the dominant SUMO species and the majority of target proteins are exclusively modified by SUMO 1 [149]. However, in response to cellular stress, including heat shock, oxidative stress, and DNA damage, SUMO 2/3 forms polySUMO chains, while SUMO 1 may only cap these chains [4, 140]. A study in HD found higher levels of high molecular weight SUMO 2/3 in insoluble fractions of HD affected striatum and suggested that this resulted from toxic mutant huntingtin protein inducing a cellular stress response [105]. Increased oxidative stress and DNA damage in FXTAS have been well reported [2, 117, 120, 131]. PolySUMOylation has been found to drive formation of biomolecular condensates through multivalent interactions [116], so it is plausible that the polySUMO chains found in FXTAS inclusions may be a driver of, or participate in the formation of inclusions. Since SUMO 2 aggregation appears to precede formation of canonical ubiquitin-containing inclusions, it is possible that SUMO 2 aggregation helps initiate inclusion formation.

Beyond polySUMOylation, other inclusion-enriched proteins point to a global increase in DNA damage. SUMO 2 and ubiquitin are both found at high levels in FXTAS inclusions modifying a large pool of proteins, and SUMO 2 IP was able to pull down large amounts of ubiquitin in FXTAS samples. Therefore, it is likely that mixed ubiquitin/SUMO chains are present. There have been reports that ubiquitin and SUMO 2 compete for the same modification sites on proteins, and may have opposing effects [75, 139], but SUMO 2/3 is also known to form mixed chains with ubiquitin that can act cooperatively to recruit DDR mediators to damaged sites or enhance protein degradation [4, 41]. Moreover, increased modification of proteins by SUMO-2/3 is a cytoprotective response against cell stress [86], which is clearly involved with FXTAS pathogenesis**.** p62 is also a known player in DDR response, and is known to be a cargo receptor that directs tagged proteins destined for degradation by the autophagosome and has previously been considered a major component of cytoplasmic inclusions [80, 154]. We speculate that its binding dynamics are not significantly different in the nucleus. Monomeric p62 is able to bind ubiquitinated substrates through its ubiquitin-associated domain [56], and it has been shown to actively participate in DNA damage repair, acting as a shuttle to transport modified proteins to the UPS or the autophagosome for removal [51, 143]. In addition, p62 regulation has been directly linked to calpain protease activity [20, 156], which may provide a link between the results presented here and Ca and calpain dysregulation seen previously in FXTAS [117].

Recently, we demonstrated that μ-calpain activity was chronically elevated in cortical and hippocampal tissues prepared from mice expressing *FMR1* CGG expansions repeats in the high range (premutation mice), beginning as early as age 6 weeks and reaching >2-fold that of age matched WT mice by 6 months [117]. The increased μ-calpain activity is the likely consequence of chronically elevated cytoplasmic Ca^2+^ and oxidative stress within premutation neurons [117]. Elevated μ-calpain activity in premutation brain was also associated with a 2-fold higher p25/p35 ratio, dysregulation of Cdk5, and elevated P-Ser^1981^-ATM – all by 6 months. These findings were in accord with corresponding molecular outcome measures in premutation hippocampal neurons at 7 DIV, the latter outcomes all normalized by the ER-Ca^2+^ channel inhibitor dantrolene [117]. Neuronal μ-calpain activity has been implicated as a primary mechanism regulating p62, which acts as an autophagic receptor that recognizes misfolded protein aggregates targeted for lysosomal degradation [12, 33, 95]. Adaptive autophagy in response to conditions of oxidative stress can have protective or pathological influences on neurons. Basal autophagy is important for the turnover of organelles and proteins preventing accumulation neurotoxic aggregates associated with neurodegeneration [71]. However, autophagy is vulnerable to other stress signals that can disrupt adaptive functions, with abnormal Ca^2+^/calpain regulation providing a mechanistic link between autophagy and apoptosis [103, 150], suggesting that chronically elevated Ca^2+^ and calpain activation may drive an autophagic survival response into maladaptive autophagy that contributes to neuronal death in FXTAS. Considering the significantly elevated levels of p62 and SUMO2 in the nuclear compartment and inclusions of FXTAS brains, we posit involvement of maladaptive autophagy in the neurodegenerative phase of FXTAS. This may not be overly speculative as ATM-mediated DNA repair is a consequence of p62 accumulation and has been linked to neurodegeneration [63, 81, 141].

**Fig. 7:**
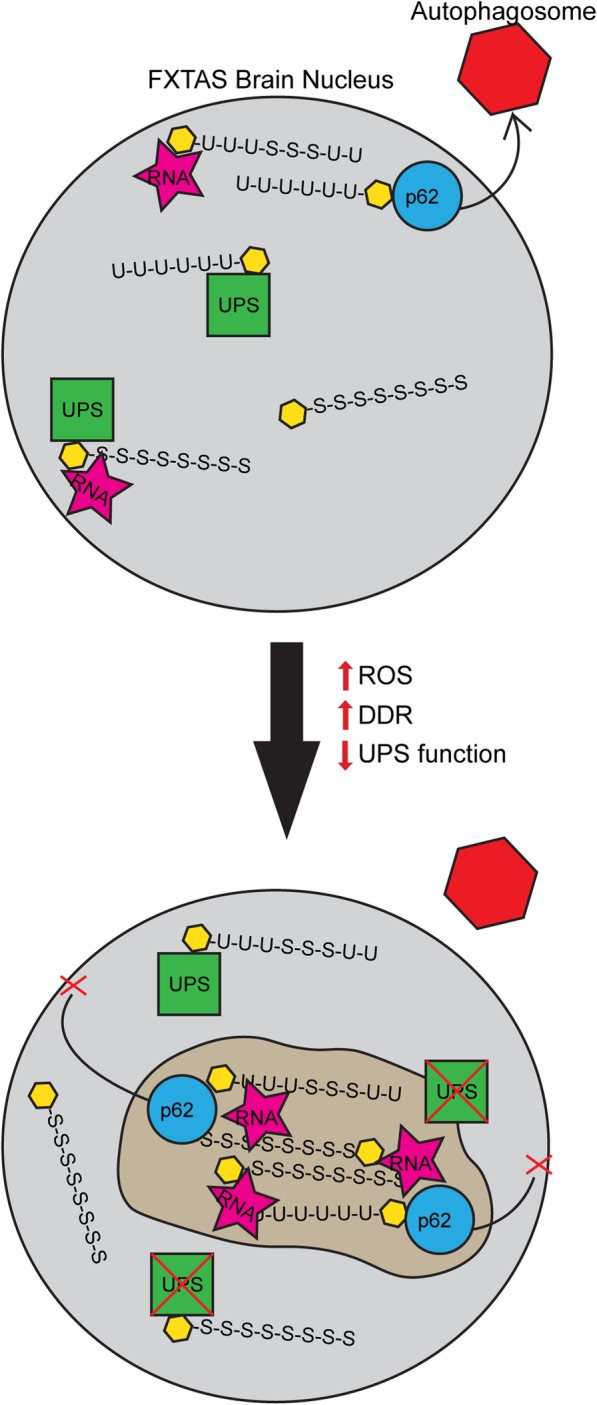
Diagrammatic representation of hypothesized FXTAS inclusion formation. Within FXTAS brain nuclei, proteins (yellow hexagons) destined for removal are tagged with ubiquitin and SUMO 2/3 chains, which are bound by the UPS for degradation. Polyubiquitinated and polySUMOylated proteins may form small aggregates with each other and/or RNA, at which point p62 will shuttle the aggregate out of the nucleus to an autophagosome for removal. Over time, as the FXTAS patient experiences higher levels of oxidative stress and DNA damage and decreased functioning of the UPS due to aging or injury, the levels of damaged/oxidized proteins and DNA damage mediators requiring removal increase. Paired with decreased UPS functionality, these proteins get tagged for removal but build up in the nucleus, aggregating with other proteins and RNA. p62 may attempt to shuttle the aggregate out to the autophagosome, but if the accumulation becomes too large, p62 has no way of shuttling the mass out of the nucleus in post-mitotic cells, resulting in an inclusion

Based on the current data, we propose a model for inclusion formation (Fig. 7) wherein a cellular stress response from a global increase in ROS and mitochondrial dysfunction [100, 117, 120], perhaps exacerbated by ROS-induced DNA damage, results in increased load of damaged/oxidized proteins as well as proteins that are involved with DDR. Ubiquitin and SUMO 2/3 target these proteins for degradation. However, if the production of these damaged and/or tagged proteins exceeds the capacity of the proteasomal machinery within the nucleus, the proteins alone or in combination with various mRNA species (e.g., *FMR1* CGG repeat mRNA) known to be present in the inclusions [135] may drive aggregation. Macroscopic aggregation would appear as an inclusion, which would not be possible for the UPS to degrade, and may even impair proteasomal function [80]. Autophagy is the normal cellular mechanism for aggregate degradation, and p62 binds the aggregation to ferry it to the autophagosome for removal [143], but beyond a certain point, the inclusion becomes too large to exit the nucleus, resulting in a chronic nuclear accumulation. The UPS system has been shown to exhibit decreased function with aging, and there has been some evidence of UPS impairment in other neurodegenerative diseases, which may explain why FXTAS symptoms and inclusion formation are most apparent at a later age [9, 66, 84, 99]. In addition, inhibition of the UPS system has been shown to generate inclusion-like structures, which further supports the connection between inclusion formation and UPS impairment [76, 77, 110].

One interesting result of the current study was the finding of extremely low levels of FMRpolyG either within inclusions or in FXTAS nuclei. It has been proposed that FMRpolyG, in combination with proteins such as LAP2β and TRA2A, aggregate to form FXTAS inclusions [15, 19, 118, 125, 137]. Our study is the first to identify endogenous FMRpolyG in FXTAS patient samples by direct protein sequencing. However, although the current study did detect minute quantities of FMRpolyG in inclusions and in SUMO 2/3 IP-enriched samples, the levels render it unlikely that FMRpolyG is a main driver or material participant in the formation of FXTAS inclusions. Moreover, FMRpolyG was undetectable in total nuclear samples, indicating that it exists at extremely low endogenous levels. Although LAP2β and TRA2A were also detected in inclusions, they were also found at very low levels (Additional file 1). LAP2 β is estimated to be present at about the 0.02-0.04% level, whereas TRA2A is present only at ~0.0002% (2 ppm) and is actually about 10- to 15-fold lower in the inclusions than in the surrounding nuclear milieu. Therefore, although LAP2β AND TRA2A have been observed by others to co-localize with FMRpolyG in FXTAS inclusions [19, 125], it is unlikely that these proteins substantively contribute to inclusion formation.

Part of the difficulty in assessing the involvement of potential inclusion proteins such as FMRpolyG, LAP2 β, and TRA2A by immunocytochemistry is that the method, though great for localization of proteins, is not a sound method for quantification, since antibody staining efficiency and methodology can have large effects on the intensity of staining. LAP2β and TRA2A are cases in point, as previously published immunofluorescence studies have demonstrated the presence of these proteins in inclusions, yet the actual levels of these proteins in inclusions are extremely low. As another example, we performed immunofluorescence on FXTAS inclusions using previously published antibodies against FMRpolyG [15], and we found highly variable staining patterns in inclusions, varying from very faint, circumscribed, and very bright, all on the same slide (Additional file 1: Figure S8a). Thus, although the RAN translation pathogenic model cannot be ruled out by the current results, the current findings underscore the need for more studies that involve endogenous expression rather than *in vitro*, induced, or high-expression models. Finally, the current study also shows that co-localization between the *FMR1* gene and FXTAS inclusions does not consistently occur, which rules out DDR as a response to damage at the *FMR1* locus as the initiator of inclusion formation.

The current study used a novel method to purify endogenous FXTAS inclusions from human brain whereby the intrinsic autofluorescence of the inclusions was used as a means for their separation, via preparative FACS, from other nuclear particles and organelles (e.g., nucleoli). The vast majority of studies of the protein composition of inclusions and other nuclear or cytoplasmic aggregates in neurodegenerative disorders, including our own [6, 58], have relied on immunocytochemistry and other staining methods [1, 3, 31, 65, 73, 98, 110]. Such methods depend not only on the uniform presence and accessibility of the specific proteins being probed, but also on the properties of antibody sensitivity and specificity. Moreover, many studies have utilized *in vitro* induction of inclusion-like bodies, which may not fully recapitulate endogenous conditions [77, 91, 97, 133, 153, 157]. Among the few studies that have purified endogenous inclusions from human patients for proteomics, methods for purification have relied on immunolabeling or sequential extraction by detergents or chaotropes for which it was assumed that the most insoluble fraction was purified inclusion [37, 62, 93, 101, 112, 128, 144]. These methods introduce bias if antibodies were used, and insoluble fractions may or may not contain a truly pure population of inclusions. The method used in this study avoids these areas of bias by utilizing autofluorescent properties of FXTAS inclusions for FACS. As a concrete example of this distinction, the autofluorescent inclusions are quite distinct from the other major intranuclear organelle, the nucleolus, which is completely non-autofluorescent (Additional file 1: Figure S8b).

The discovery of autofluorescence in FXTAS inclusions allows us to more specifically identify and isolate inclusions, but also calls for more stringent guidelines when examining inclusions by immunofluorescence. This finding does not necessarily nullify past immunofluorescence results on inclusions, but such results should now be reassessed using immunofluorescence strategies that take autofluorescence into account. Such strategies are already in use in the field of NIID, which noted autofluorescent inclusions at certain wavelengths [70, 110]. More detailed recommendations will be published in a separate technical paper. However, in brief, FXTAS inclusion autofluorescence interferes most strongly in the green, yellow, and orange spectrum. Since the blue range of the spectrum is usually reserved for DAPI staining, efforts should be made whenever possible to perform inclusion immunofluorescence in the far-red spectrum. There are still low levels of autofluorescence in that spectral region. Therefore, slides with secondary-only antibody staining should be imaged using the same settings as those with primary antibody staining of inclusions and provided for comparison. If it is necessary to perform immunofluorescence at a wavelength where autofluorescence is substantial, the antibody used must first be verified to have sufficient sensitivity and specificity to overcome autofluorescence.

The use of an autofluorescence-based method of purifying inclusions is novel; therefore, it is important to verify that what is collected from FACS is a substantially pure population of inclusions. In the current instance, microscopy of the sorted inclusions verified that their autofluorescence profile was the same as that of inclusions *in situ* (Fig. 1 c). Moreover, western blot and immunofluorescence analyses verified that two of the newly-identified inclusion proteins that were most enriched by MS are increased both in FXTAS nuclei and in FXTAS inclusions (Fig. 6, Additional file 1: Figure S7). Interestingly, several of the proteins found to be enriched in FXTAS inclusions correspond to proteins found in the inclusions of other neurodegenerative disorders. Two of the FXTAS inclusion proteins with greatest enrichment – ubiquitin and p62 – have previously been well-documented. Ubiquitin is a component in some forms of FTD, HD, some forms of spinocerebellar ataxia (SCA), PD, ALS, dentatorubral-pallidoluysian atrophy (DRPLA), and NIID [11, 28, 49, 70, 72, 87, 123]. p62 has also been found in other inclusion disorders, including FTD, PD, and AD [49, 88, 154]. A third protein enriched in FXTAS inclusions, MLF2, has previously been found to co-aggregate with p62 and poly (gly-ala) in mice, and it alleviated poly-Q associated toxicity in Drosophila and rat models [36, 124]. The presence of MBP enrichment in FXTAS inclusions is not well understood, as MBP is traditionally known to be a cytoplasmic oligodendrocyte protein associated with myelination. However, variant forms of it have been found to inhabit oligodendrocyte nuclei [108] and to play unrelated functional roles in astrocytic and neuronal nuclei [74]. Regardless, it has previously been observed as a FXTAS inclusion protein [58] and further investigation into its role is warranted. In addition, the functional families of proteins found enriched in inclusions are similar to what has been seen in other types of inclusions. RNA binding proteins, and especially hnRNPs, have previously been detected in other types of inclusion disorders, as well as chaperones, particularly heat shock proteins [21, 24, 34, 65, 92, 111]. The presence of proteasomal proteins in FXTAS inclusions is not surprising as a connection between the UPS and inclusions has long been suspected.

Aside from comparison to inclusion composition in other disorders, the FXTAS inclusion composition was also compared to that of lipofuscin to rule out the possibility that lipofuscin was a significant component of sorted FXTAS inclusions. Lipofuscins are intracytoplasmic, perinuclear pigment granules that are composed of the residue of lysosomal degradation of aggregated/damaged proteins. It accumulates in normal aging human brain, and autofluoresces throughout a broad range of wavelengths. The main difference in inclusion and lipofuscin autofluorescence is in the far-red wavelengths, where lipofuscin autofluorescence is significantly more intense than inclusion autofluorescence (Additional file 1: Figure S1). It is unlikely that sorted inclusions contain a significant component of lipofuscin for the following reasons: (i) the nuclear isolation step should have largely eliminated extranuclear cell debris, which would include the cytoplasmic lipofuscin; (ii) the FACS sorted fraction from the nuclear isolates was only present in the FXTAS cases, not in controls, whereas lipofuscin is present in controls as well as FXTAS cases; (iii) the sorted inclusions were additionally viewed by microscopy to ensure that a minimal proportion of sorted particles were brightly fluorescent in the far-red wavelength (Fig. 1d); and (iv) we compared lipofuscin protein components as identified by MS in a previous publication to the composition of the FXTAS inclusions determined in the current study [106]. Out of 49 identified major lipofuscin proteins, 7 were not found in our dataset, 7 were found at extremely low levels (<0.005%), and almost all the proteins that were found in both datasets were not enriched in FXTAS inclusions over total nuclear samples.

The current investigation has some limitations that bear consideration. First, although the purification process for inclusions is robust and more specific than other inclusion isolation processes in the field, we estimate that as much as 10–20% of noninclusion material may remain in the final preparations. This could allow some proteins that are not within the inclusion to be detected by MS, although the comparison to accompanying nuclear samples – establishing levels of enrichment – should help to mitigate this problem. Second, due to the need for long sorting times and amounts of brain tissue required to isolate sufficient numbers of inclusions for MS, only two FXTAS patients were analyzed. Third, a limitation of the MS analysis is that the quantification and percentages of proteins composing a population is limited by the population of proteins detected. Therefore, if there are undetectable proteins due to insufficient solubility, lack of available tryptic cut sites, or unforeseen pos-translational modifications, these would not be represented in the protein population. Regarding FMRpolyG, it has been reported that solubilization may be challenging. However, we used mild heating in a strong detergent buffer, the same strength as that used in a previous western blot of FMRpolyG [137], as well as formic acid; we did not observe any precipitate formation. Previously, only two western blots for FMRpolyG on native human brain tissue have been published [125, 137], and both utilized thermal denaturation by boiling the tissue samples. It may be that solubilization problems arise due to heat-dependent denaturation/aggregation, as has been established previously [8, 40, 122, 138, 148].

## Conclusions

We have performed a detailed analysis of the protein composition of isolated FXTAS inclusions using their distinct autofluorescent properties as a means for preparative FACS-based purification. Although the current study has helped to refine our understanding of the role played by inclusions in FXTAS pathogenesis, further investigation into the role of SUMO2/3 in FXTAS pathogenesis is warranted, as well as a re-investigation of the roles of FMRpolyG in this disorder. Finally, we wish to point out that models proposed for mRNA sequestration remain viable [60, 115, 126, 127, 130], and these models/mechanisms should be investigated further.

## Additional file

Additional file 1: This file contains additional methods, supplementary figures, figure legends, and tables in support of the main text. (DOCX 5244 kb)

Additional file 2: This file contains raw data for the two MS analyses in the tabs labeled “Protein groups”; analyzed data to calculate relative percent abundances of identified proteins in the tabs labeled “Relative abundance”; and a list of the 176 proteins found enriched in FXTAS inclusions and used for Fig. 3 in the tab labeled “176 enriched inclusion proteins. (XLSX 2720 kb)

## Data Availability

The proteomics datasets generated and analyzed in the current study are available in the MassIVE repository (massive.ucsd.edu) under ID MSV000084199.

## Abbreviations

AD: Alzheimer’s disease
ALS: amyotrophic lateral sclerosis
BSA: bovine serum albumin
CBR1: carbonyl reductase 1
CERAD: Consortium to Establish a Registry for Alzheimer’s Disease
CRYAB: αβ-crystallin
DDA: data-dependent acquisition
DDR: DNA damage response
DM: myotonic dystrophy
DRPLA: dentatorubral-pallidoluysian atrophy
FACS: fluorescence activated cell sorting
FISH: fluorescence in situ hybridization
FTD: frontotemporal dementia
FXS: fragile X syndrome
FXTAS: Fragile X-associated tremor/ataxia syndrome
HCD: high energy collisional dissociation
HD: Huntington’s Disease
hnRNP: heterogeneous nuclear ribonucleoprotein
hpCGG: high-premutation CGG
IDH2: isocitrate dehydrogenase
IP: immunoprecipitation
LAP2β: lamina-associated polypeptide 2 beta
LC-MS/MS: liquid chromatography/tandem mass spectrometry
MBP: myelin basic protein
MLF2: myeloid leukemia factor 2
MS: mass spectrometry
NHEJ: non-homologous end joining
NIH: National Institutes of Health
NIID: neuronal intranuclear inclusion disease
PARP1: poly [ADP-ribose] polymerase 1
PD: Parkinson’s disease
PPIA: peptidyl-prolyl cis-trans isomerase A
PSP: progressive supranuclear palsy
RAN: repeat-associated non-ATG
SCA: spinocerebellar ataxia
SQSTM1: sequestosome1
STED: stimulated emission depletion
SUMO 2: small ubiquitin-related modifier 2
TBS: tris buffered saline
TEAB: triethylammonium bicarbonate
TRA2A: transformer-2 protein homolog alpha
UCD: University of California, Davis
WT: wildtype
XRCC5: x-ray repair cross-complementing protein 5
XRCC6: x-ray repair cross-complementing protein 6
