# Supplementary material for: Composition of the Intranuclear Inclusions of Fragile X-associated Tremor/Ataxia Syndrome
Source: Acta Neuropathol Commun. 2019 Sep 3;7:143. doi: 10.1186/s40478-019-0796-1 (PMC6720097; doi:10.1186/s40478-019-0796-1)
Supplement: Supplementary file 1 — This file contains additional methods, supplementary figures, figure legends, and tables in support of the main text. (DOCX 5244 kb) [file 40478_2019_796_MOESM1_ESM.docx]

**Supplementary Materials**

**Supplementary methods**

**Brain nuclei slide preparation.** Slides of brain nuclei were prepared using the methods described in Iwahashi et al. [58]. Pulverized fresh frozen brain was loosely Dounce homogenized in HBC homogenizing buffer (0.32 M sucrose, 50 mM Tris, 5 mM EDTA, 17 µg/ml phenylmethylsulfonyl fluoride (PMSF), Complete protease inhibitor Roche cat #11697498001, pH 7.4) then filtered successively through 500 µm and 100 µm nylon mesh before centrifuging at 1,500 RCF for 10 min at 4 °C. Pelleted nuclei were then washed three times in HBC before resuspending in 1 ml to 2 ml of BCC (20 mM HEPES, 400 mM NaCl, 1 mM DTT, 1 mM EDTA, 1 mM EGTA, Complete protease inhibitor, pH 7.4), and spreading onto SuperFrost Plus slides and fixing in 70% v/v methanol for 15 min at room temperature.

**Fluorescence microscopy.** All images taken using an upright microscope were taken on a Leica DM5500B. Gain was set at 2.3x and A4, L5, Y3, and Y5 filter cubes were used to observe the images. Images were saved as TIF files and analyzed using ImageJ software. For images utilizing autofluorescence to observe inclusions, including those with and without antibody staining, exposure levels were kept constant and are displayed on all figures, and no post-processing was done (including any to adjust brightness or contrast) to ensure that comparisons could be made from image to image. For images pertaining to DNA FISH and inclusion colocalization, post processing to adjust brightness and contrast was used to allow clear visualization of the FISH signal.

**DNA fluorescence in situ hybridization (FISH).** Three human BAC clones (RP11-37P24, RP11-949I9, and RP11-161L9) spanning approximately 200 kb surrounding and including the *FMR1* gene were obtained from Children’s Hospital Oakland Research Institute and expanded under chloramphenicol resistance. BAC DNA was isolated and clone identities were verified by restriction enzyme digest followed by gel electrophoresis. The UCSC Genome Browser and RepeatMasker were used to exclude repetitive and low complexity regions within the 200 kb region, and Taq PCR primers were designed to generate amplicons 200 to 330 base pairs in size within the remaining non-repetitive regions. Using the BAC clone DNA for starting material, PCR and gel electrophoresis verification was conducted on each primer set, and primers that were nonspecific or failed to amplify were removed from further use. In total, 119 primer sets amplified strongly and specifically, spanning approximately 30 kb of DNA (Fig. S9). After primers were verified, biotin-labeled PCR amplicons were generated using a modified PCR protocol where the nucleotide mixture added to the PCR reaction contained a 1:1 ratio of dTTP and biotin-11-dUTP (NEB cat #R0081). Gel electrophoresis was performed on the labeled PCR product alongside unlabeled PCR product to verify that the product size was as expected and that biotin labeling had occurred, resulting in a band shift upwards. The FISH probe was then purified using the Zymo DNA Clean and Concentrator (Zymo cat #D4013).

Approximately 0.3-0.5 µg of probe per slide was precipitated using 2.5 volumes of ethanol and resuspended in hybridization buffer [50% formamide, 40% dextran sulfate, in 2x saline sodium citrate (SSC) buffer]. Cells were permeabilized using PBS + 0.5% Triton X-100 at room temperature for 15 min and incubated in denaturation solution (70% formamide in 2x SSC) at 72 °C for 3 min. Cells were permeabilized once more in 0.7% Triton X-100/0.1 M HCl for 10 min on ice and in 10 mM HCl for 30 min at room temperature before treating with 0.1 µg/µl of RNase A for 1 hr at 37 °C. Cells were dehydrated for 5 min each in 70%, 85%, and 100% ethanol. Probe was added to the slide, covered with a coverslip and sealed with rubber cement, and the slide was heated for 4 min at 85 °C before slides were incubated overnight at 37 °C in a humid chamber. Coverslips were removed, and slides were washed successively three times each in wash A (50% formamide in 2x SSC at 45 °C) and wash B (0.1x SSC at 60 °C). Cells were then submerged in blocking solution (3% BSA, 0.5% Tween-20, in 4x SSC) and incubated at 37 °C for 30 min, followed by a 1 hr incubation at 37 °C in detection solution (1% BSA, 0.5% Tween-20, in 4x SSC) containing Streptavidin bound to Alexa-555 (Invitrogen S32355) at a 1:200 dilution. Cells were then washed three times in wash C (0.5% Tween-20 in 4x SSC at 45 °C) before staining with DAPI and mounting with Prolong Diamond Antifade Mountant.

**DNase, RNase, and Protease treatment of isolated inclusions.** Inclusion-enriched fractions were equally separated into 4 aliquots and centrifuged at 2,000 RCF for 10 min at 4 °C. Each aliquot was resuspended in 500 µl of a designated treatment: 1) BDC + 0.1% NP-40; 2) 100 U of DNase I (Thermo Scientific cat #EN0521) + 0.1% NP-40 in BDC; 3) 10 µl of RNase A/T1 (Thermo Scientific cat #EN0551) + 0.1% NP-40 in BDC; and 4) 200 µg/ml of proteinase K (Thermo Scientific cat #EO0492) + 0.1% NP-40 in BDC minus Complete protease inhibitor. All samples were incubated on a rotator at 37 °C for 2 hr before being centrifuged at 2,000 RCF for 5 min at 4 °C, then spread and fixed onto slides using 70% v/v methanol. Slides were air dried then immediately mounted with coverslips using SlowFade Antifade Mountant and visualized immediately.

**Nuclear Isolation.** Fresh frozen human brain tissue from the frontal cortex was pulverized in liquid nitrogen, and all buffers were prepared the day before use. 0.5 g to 1 g of tissue was Dounce homogenized using a loose pestle in NI buffer (0.32 M sucrose, 1 mM KH_2_PO_4_, 1 mM MgCl_2_, 0.25% Triton X-100, 0.1mM PMSF, pH 6.5) on ice for 20-30 strokes. Suspension was filtered successively through 500 µm and 100 µm nylon mesh, then centrifuged at 850 RCF for 10 min at 4 °C, and the pellet was resuspended in 10ml of NII (0.32 M sucrose, 1 mM KH_2_PO_4_, 1 mM MgCl_2_, 0.25% Triton X-100, 0.1 mM PMSF, pH 6.5). No sample was allowed to be submerged in NI for longer than 20 min. Samples were centrifuged again at 850 RCF for 10 min, resuspended once more in 10ml of NII, then centrifuged for 8 min at 650 RCF. Pellet was resuspended once more in 4ml of NII. The rest of the sample was mixed thoroughly with 25 ml of NIII (2.39 M sucrose, 1 mM KH_2_PO_4_, 1 mM MgCl_2_, 0.1 mM PMSF, pH 6.5) before ultracentrifugation in a swing bucket rotor at 63,600 RCF for 2 hr at 4 °C. Supernatant was removed without disturbing the nuclear pellet, and the pellet was submerged in 1ml of BCC for 20 min on ice before the pellet was resuspended thoroughly by pipetting and centrifuged at 2,000 RCF for 5 min at 4 °C Pellets from the same patient were then pooled and resuspended in 1.7 ml of BCC with the addition of RNase inhibitor and stored overnight at -80 °C.

**Fluorescence-activated cell sorting (FACS) of inclusions.** The Astrios is equipped with high-powered violet (405 nm), blue (488 nm), yellow (561 nm), and red (640 nm) lasers and can detect up to 18 separate emission colors. As observed by fluorescence microscopy, the inclusions present in FXTAS tissue homogenates were small, relatively homogenous in size and primarily exhibited green autofluorescence (500-565 nm) following 488 nm laser excitation. The autofluorescence of samples from control and FXTAS individuals were assessed on the Astrios using each laser line and across various detectors assigned to each laser to determine the strongest detection signals. Similar to immunofluorescence microscopy, the inclusions in FXTAS tissues exhibited readily detectable green to orange fluorescence but showed little emission in red-shifted wavelengths. These green fluorescent particles were not apparent in similarly prepared samples from control tissues. In standard antibody-based immunolabeling strategies for protein detection, autofluorescence is viewed as a source of contaminating background light that partially masks signal detection. In some biological systems, however, a lack of readily-available antibody labeling tools hampers the application of standard imaging and cytometric approaches. In these cases, it is possible to use the intrinsic, native autofluorescence to detect and enrich for specific biological particles, such as plant tissues, microorganisms and sub-cellular particles that are important in many fields of plant, marine and cell biology [119,147,27]. The autofluorescence of small particles, such as marine bacteria, microparticles, and protein aggregates may be dim, but detectable over the standard optical background from debris. It is standard practice in the field of flow cytometry to detect small sub-cellular particles using logarithmic scaling on the detectors assigned to laser light scatter measurements [138]. Accordingly, we used logarithmic scales to discern small protein inclusions from debris artifacts introduced in the sample buffer. We then removed larger aggregates by plotting the duration of 90° laser light scatter to remove objects with markedly increased laser dwell rates relative to the shorter transit times of single particles. Under these Astrios instrument conditions, we compared the strength of the autofluorescence signals in FXTAS and control samples across several detectors. We noted that the strongest fluorescence signal was measured in the green detector from 488 nm laser excitation, but this signal was markedly diminished in >670 nm wavelengths when subjected to 488, 561, or 640 nm laser excitation.

**FMRpolyG construct generation.** To strongly drive stable FMRpolyG production, pBR-CMV-5ATG-63-EGFP plasmid (Fig. 5a) was generated to produce FMRpolyG containing 63 CGG repeats fused to EGFP from a strong CMV enhancer plus promoter and a canonical ATG start of translation rather than the native ACG. The CMV enhancer and promoter from pRL-CMV (Promega) and a 2.3 kb fragment containing a low copy number origin of replication and ampicillin resistance gene from pBR322 (NEB) were used as the vector backbone. PCR generated *FMR1* sequence containing the 5´ untranslated region (UTR) and Exon 1 was ligated to oligos containing the first three base pairs of Exon 2 followed by several translational stop codons in frame with FMRP. EGFP followed by a poly(A) signal were added in frame with FMRpolyG.

SK-N-MC cells (SK; neuroepithelial origin; ATCC) were grown to ~80% confluency in DMEM supplemented with 10% fetal bovine serum and ~100 U/mL penicillin and 100 ug/mL streptomycin. Cells were then trypsinized and transiently transfected with plasmid DNA using the Neon Transfection System and antibiotic-free DMEM (Pulse voltage: 1300 v, Pulse width: 30 ms, Pulse number: 1). Forty-eight hours after transfection, floating and adherent cells were harvested by scraping, washed with PBS, spun down, and flash frozen in liquid nitrogen. Cell pellets were stored at -80 °C.

**SUMO 2/3 Immunoprecipitation.** 6 g of pulverized, fresh frozen frontal cortical tissue was processed from two FXTAS patients and two age-matched controls. Nuclear isolation was performed as described previously, except 1 mM EDTA, 1 mM EGTA, and 50 mM N-ethylmaleimide (NEM) was added to buffers. Isolated nuclei were lysed in lysis buffer (50 mM Tris, pH 7.5, 50 mM NaCl, 0.5% sodium deoxycholate, 1 mM EDTA, 0.25% Triton, 5% SDS, 30% glycerol, complete protease inhibitor), then run through a 27-gauge needle until viscoelasticity from nucleic acids was reduced. An aliquot of nuclear lysate was reserved for western blot. The rest of the nuclear lysate samples were heated to 50 °C for 5 min, then diluted with IP buffer (50 mM MOPS buffer, 150 mM NaCl, 10 mM Na_2_HPO_4_, complete protease inhibitor) by 10x to reduce final detergent concentrations to 0.5% SDS, 0.025% Triton, and 0.05% sodium deoxycholate. Lysates were centrifuged at 16,000 RCF for 20 min at 4 °C to pellet insoluble material, and supernatant was used for downstream experiment. BCA assay was performed on lysate, and 3 mg of each sample was incubated with 20 µl of SUMO 2/3 antibody (100-200 µg) for 1 hr at 4 °C with rotation. 750 µl of Protein G Magnetic Beads (NEB cat #S1430S) (enough to bind 187.5-225 µg of antibody) per sample were washed three times with IP buffer before incubating with lysate-antibody mixture for 3 hr at 4 °C with rotation. Beads were magnetized, and supernatant was saved to be run on western blot as unbound sample. Beads were washed three times in wash buffer (BCC buffer + 0.2% NP-40), and an aliquot was taken to be run on western blot as IP elute. IP elute aliquot used for western blot was eluted twice in urea elution buffer (7 M urea, 20 mM Tris, pH 7.5, 100 mM NaCl) for 30 min each at room temperature. The rest of the beads were washed three times in 50 mM TEAB buffer before on bead digestion.

**LC-MS/MS Data Analysis.** Raw DDA files were searched with Andromeda in MaxQuant (version 1.6.5.0) using default Orbitrap settings. Briefly, a target-decoy search strategy was utilized against a human protein sequence database (downloaded February 5, 2018 from Uniprot.org) consisting of 71,617 protein sequences amended with 48 potential contaminants from the cRAP database of common laboratory contaminants ([www.thegpm.org/crap](http://www.thegpm.org/crap)), FMRpolyG or FMRpolyG-GFP sequence, and an equal number of reverse decoys. Identifications were made at 1% protein and peptide FDR with match between runs and second peptides enabled. Searches were configured for trypsin allowing for two missed cleavages and carbamidomethylation of Cys as a fixed modification (except where alkylation not performed). Up to five variable modifications were allowed per peptide including oxidation of Met and N-terminal acetylation. Instrument parameters and match tolerances were set to Orbitrap defaults. Label-free quantitation was performed with the fast MaxLFQ algorithm and intensity-based absolute quantification (IBAQ) using unique and razor peptides (not containing variable modifications) with a requirement for two shared peptides and large LFQ ratio stabilization enabled.

MaxQuant data output was loaded into Perseus version 1.6.0.2 for further processing and statistics. Proteins designated as reverse, contaminant, or only identified through a post-translational modification site were removed from further processing. For identification, proteins required identification by at least 1 nonredundant peptide (unique or razor) with MS/MS identification or matching. For quantitation, normalized IBAQ protein intensities were used to calculate percent protein composition within each sample. R was used to filter out proteins according to abundance and enrichment (see figure legends for details). Scaffold version 4.8.4 was used to visualize FMRpolyG sequence and spectral data.

**Online Resource 1**

Raw data for the two MS analyses are provided in the tabs labeled “Protein groups”. Analyzed data to calculate relative percent abundances of proteins identified are provided in the tabs labeled “Relative abundance”. The 176 proteins found enriched in FXTAS inclusions and used for Fig. 3 are provided in the tab labeled “176 enriched inclusion proteins”.

**Supplementary figure/table legends**

**Figure S1:**

**a: FXTAS inclusions display quantifiably different autofluorescence spectral properties compared to lipofuscin.** Scoring of inclusions versus lipofuscin autofluorescence intensities at various wavelengths identifies significant differences between FXTAS inclusion and lipofuscin autofluorescence. FXTAS isolated brain nuclei slides stained only with DAPI were analyzed on a stimulated emission depletion (STED) microscope for autofluorescence excitation/emission spectra. Inclusions were found to have maximal excitation/emission at 490 /560 nm, with an emission range from 475 nm to 740 nm, where emissions above 590 nm gradually taper off in intensity (top two images). Emissions below 475 nm could not be assessed because the confocal laser emission line was at 470nm. Autofluorescent lipofuscin particles on the same slide show similar patterns of autofluorescence, except with higher intensities of emission in far-red wavelengths. Orange arrows denote the object that is being measured. Mean autofluorescence intensities were measured for inclusions from four different FXTAS patients along with lipofuscin also found in the same images. Lipofuscin displays significantly higher autofluorescence levels at both the 545 nm and 620 nm wavelengths.

**b: FXTAS brain samples produce a diffuse band of particles at a density of ~1.3 g/ml.** FXTAS isolated brain nuclei are treated with tight Dounce homogenization, a stringent DNase I treatment, then fractionated on a sucrose gradient running from 2.6 M to 2.2 M sucrose. FXTAS samples produce a visible diffuse band of particles at the fraction corresponding to 2.3 M to 2.4 M sucrose, or 1.29 to 1.30 g/ml. There is no visible band in control samples.

**Figure S2:**

**a**: **Western blot confirms MS results that SQSTM1 is present in FXTAS brain nuclear lysates at higher levels compared to control brain nuclear lysates.** Western blot on brain nuclear protein lysates obtained from age matched control and FXTAS human samples confirmed that SQSTM1 is found at higher levels in FXTAS patient samples compared to control samples. Western blots were performed on five matched pairs of control and FXTAS brain lysates from a total of three control and four FXTAS individuals (one representative image shown here) and densitometry was used to quantify SQSTM1 levels. The red arrow points to the approximately 62 kDa band where SQSTM1 may be found.

**b**: **Immunofluorescence for SQSTM1 localizes it to FXTAS inclusions.** Immunofluorescence for SQSTM1 reveals its presence in FXTAS inclusions. A secondary antibody only control is shown (far right) to use for comparison of SQSTM1 inclusion staining fluorescence to inclusion autofluorescence at 620 nm. No post-processing was done to alter brightness/contrast levels. Orange arrows denote inclusions.

**Figure S3:**

**Total protein for SUMO 2/3 western blot on FXTAS vs. Control human brain**

**Figure S4:**

**No significant difference was found between control and FXTAS samples for SUMO 1.** Neither FXTAS nuclear nor whole tissue protein samples showed a significant difference in SUMO 1 signal compared to control samples.

**Figure S5:**

**a: Additional images of SUMO 2/3 immunofluorescence on mouse brain**

**b: SUMO 2/3 aggregates in 11-month-old high CGG FXTAS mouse model brain are not exaggerated enough to form high density smears on western blot.** Western blot was only performed on one sample each of male hemizygous high CGG, female homozygous high CGG, and male wildtype mouse brain. Although high CGG brains may display overall slightly more SUMO 2/3 signal, compared with wild type, they do not exhibit the vibrant and dense SUMO 2/3 smear on western blot, perhaps due to the smaller size and number of SUMO 2/3 aggregates.

**Figure S6:**

**Total protein for SUMO 2/3 western blot on human brain tissue (frontal cortex) from various neurodegenerative disorders**

**Figure S7:**

**SUMO 2/3 immunoprecipitation was successfully performed on two FXTAS patient samples and two age-matched control samples.** Two FXTAS nuclear lysate samples and two age-matched control nuclear lysate samples were used for SUMO 2/3 IP. Western blot on immunoprecipitation samples confirmed successful IP. Nuclear samples (nuc) are pre-IP samples. Non-IP samples are the unbound supernatant after IP. IP elute samples are the bound and eluted samples after IP.

**Figure S8:**

**a: FMRpolyG immunofluorescence on FXTAS inclusions produces highly variable staining levels and patterns.** 8FM and 9FM FMRpolyG antibodies produced high levels of background staining, and highly variable inclusion staining intensities. All three images of FMRpolyG 9FM staining were from the same slide. 360 nm + 480 nm images demonstrate inclusion localization and autofluorescence. Orange arrows denote inclusions. The top images show weak staining, the middle images show the strong staining only in a ring around the inclusion, and the lower images show strong staining.

**b:** **Nucleoli do not autofluoresce, making autofluorescence a sound strategy for isolating inclusions away from other non-autofluorescent intranuclear organelles.** Orange arrows denote inclusions and green arrows denote nucleoli.

**Figure S9:**

Primers were verified by Taq PCR and gel electrophoresis. 127 primer sets were tested. The expected amplicon sizes were around 300bp. Primer sets which failed to amplify or were unspecific were excluded from probe generation.

**Table S1:**

**Patient information on human fresh frozen frontal cortex samples**

**Table S2:**

**Patient information on human fibroblast and lymphocyte samples**

**Table S3:**

**Sample information on mouse fresh frozen cortex samples**

**Supplementary figures**

**Fig. S1**


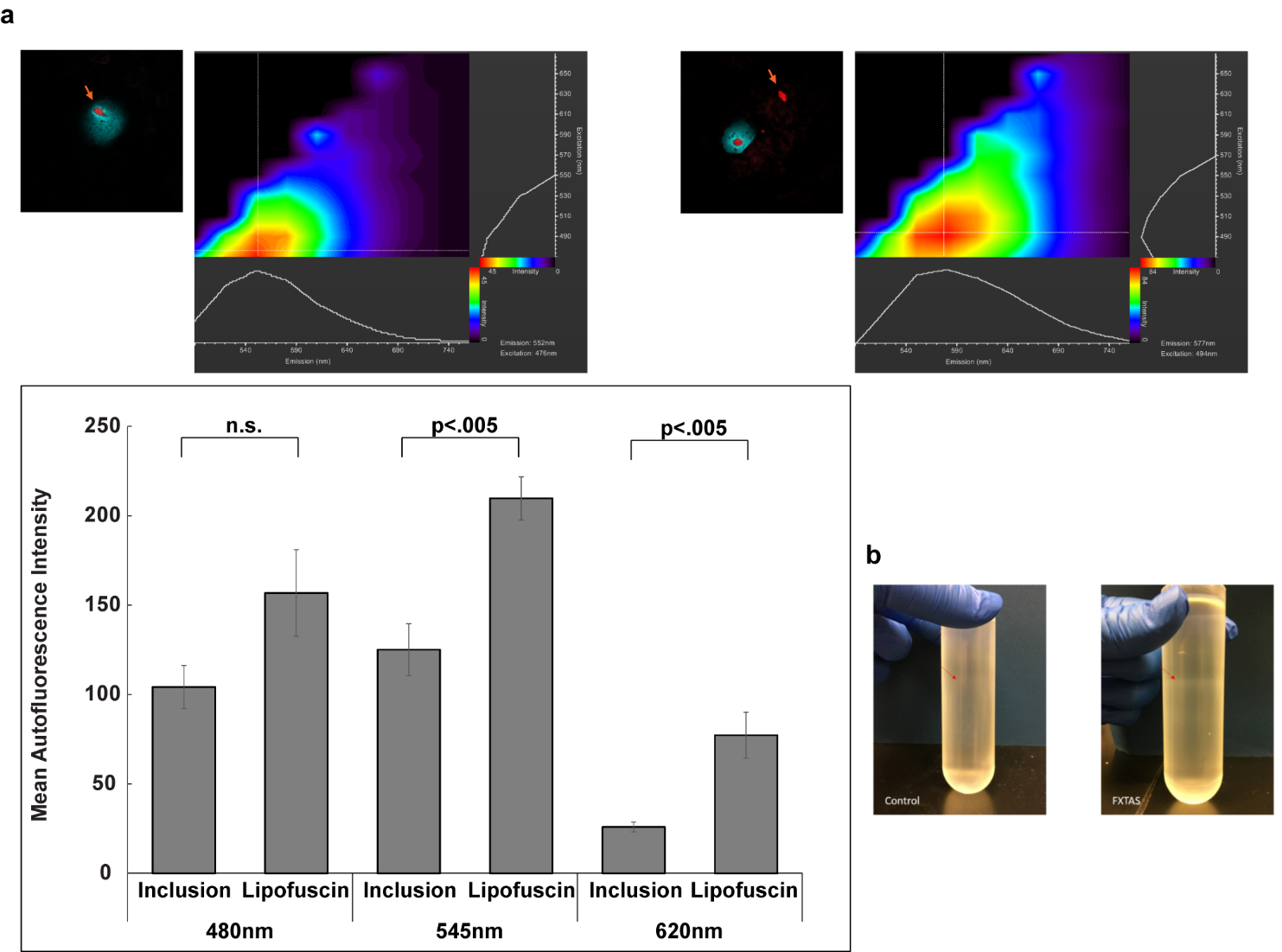


**Fig. S2:**

**
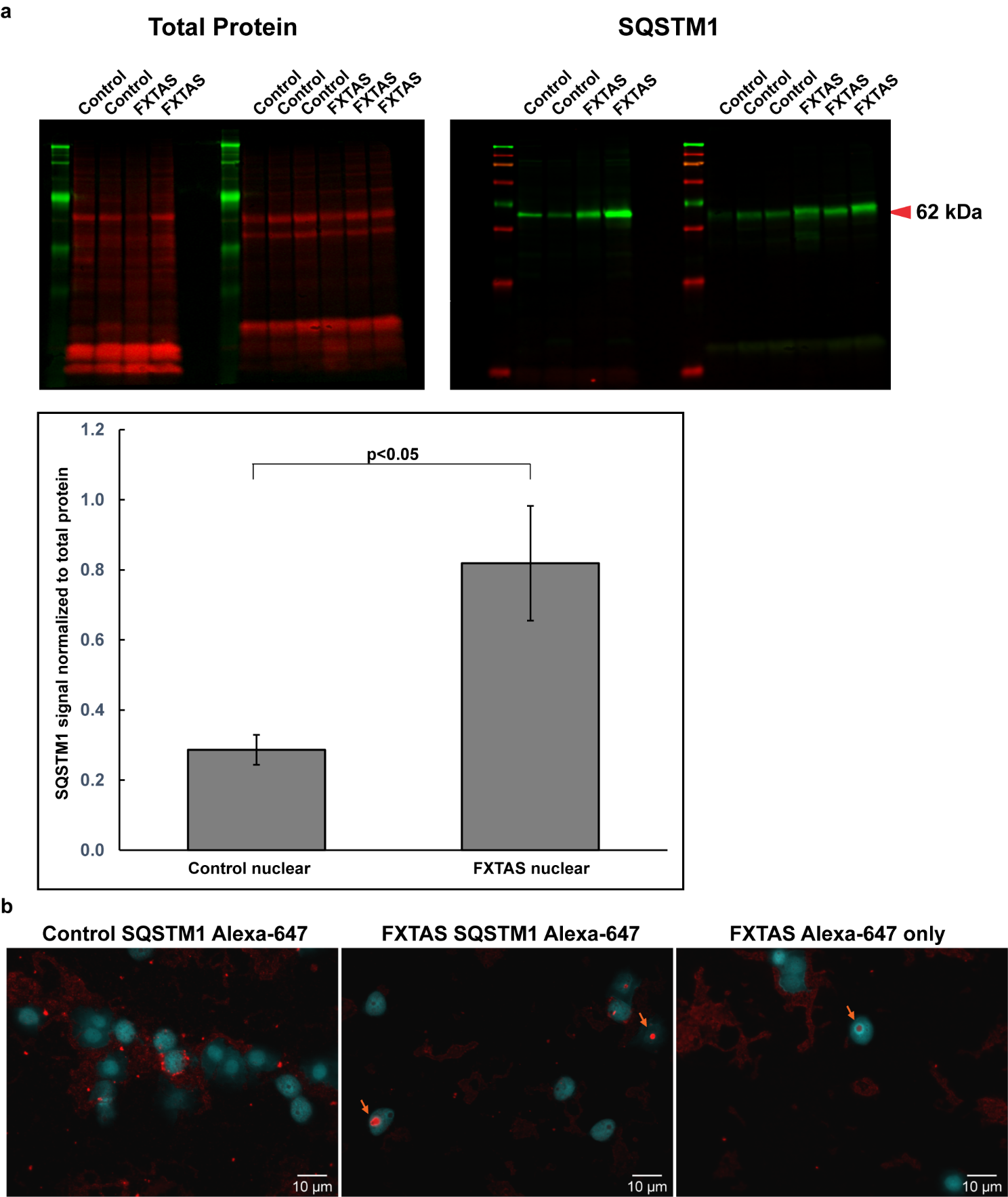
**

**Fig. S3:**


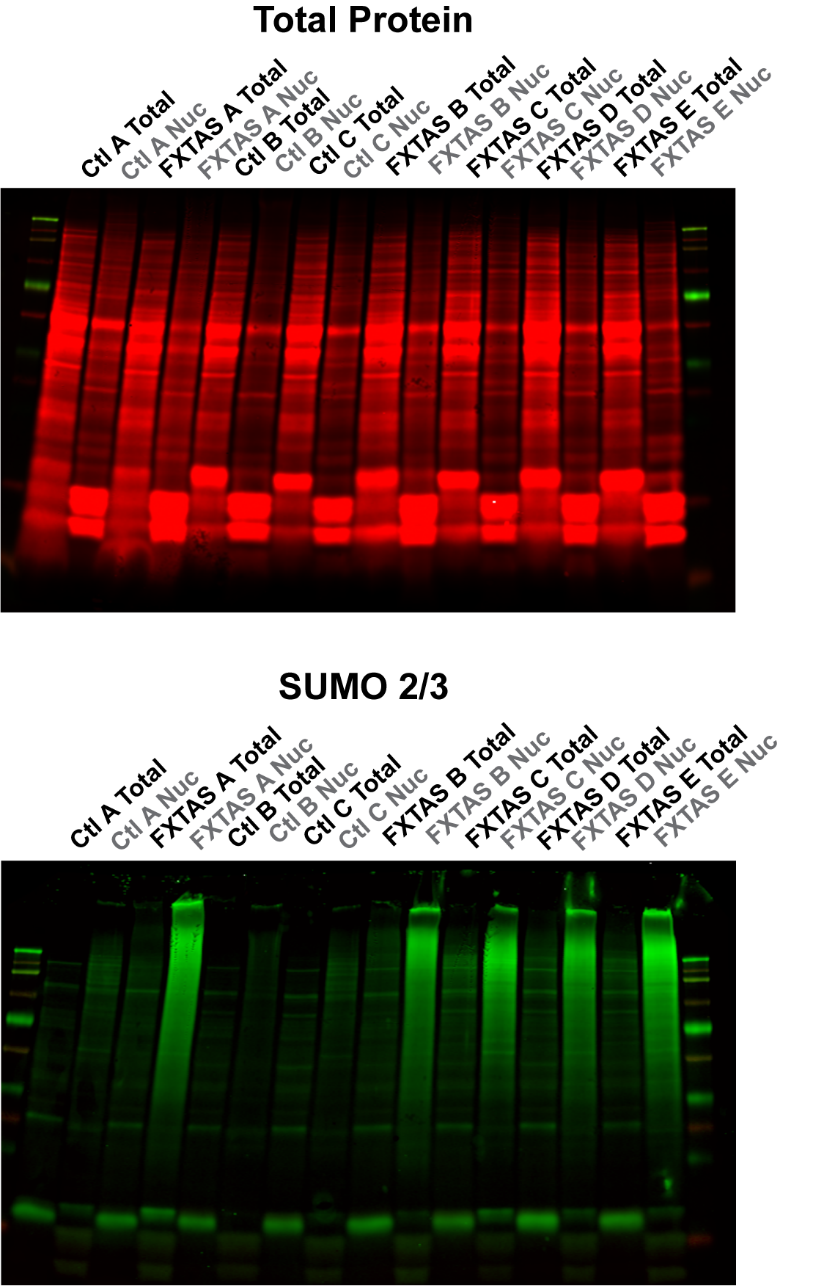


**Figure S4:**


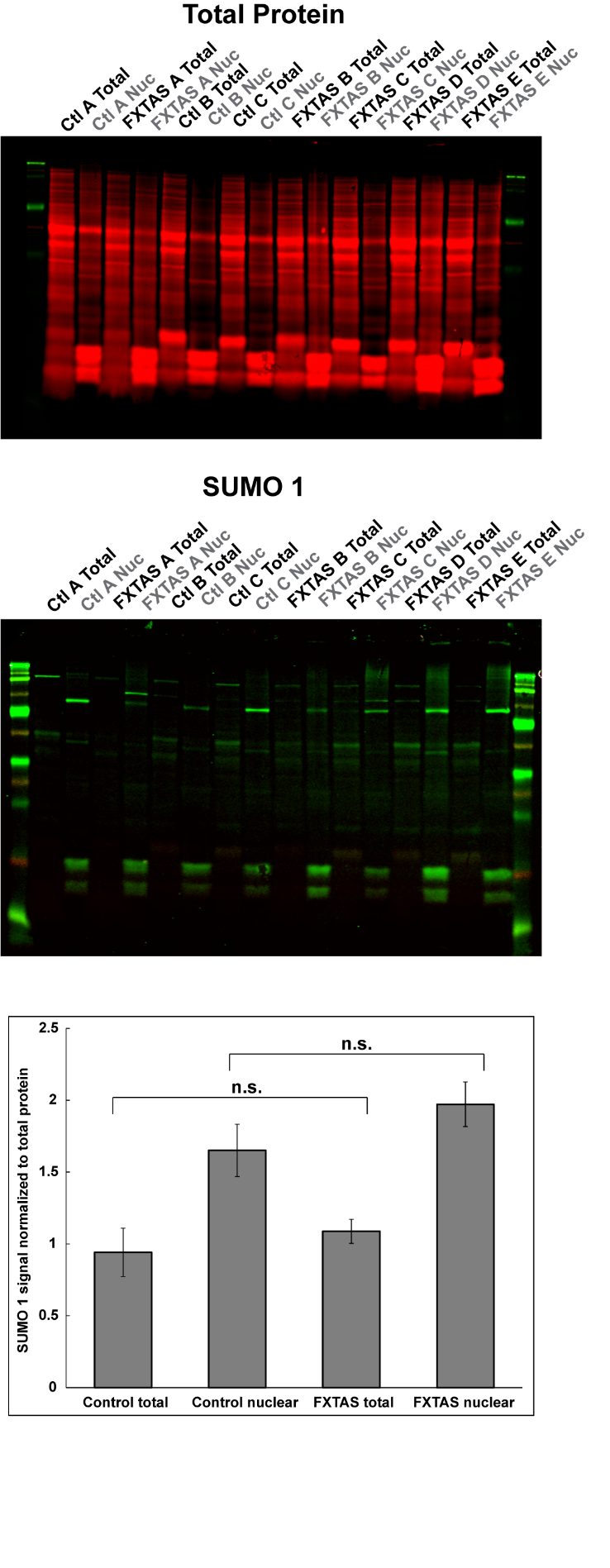


**Fig. S5:**

**
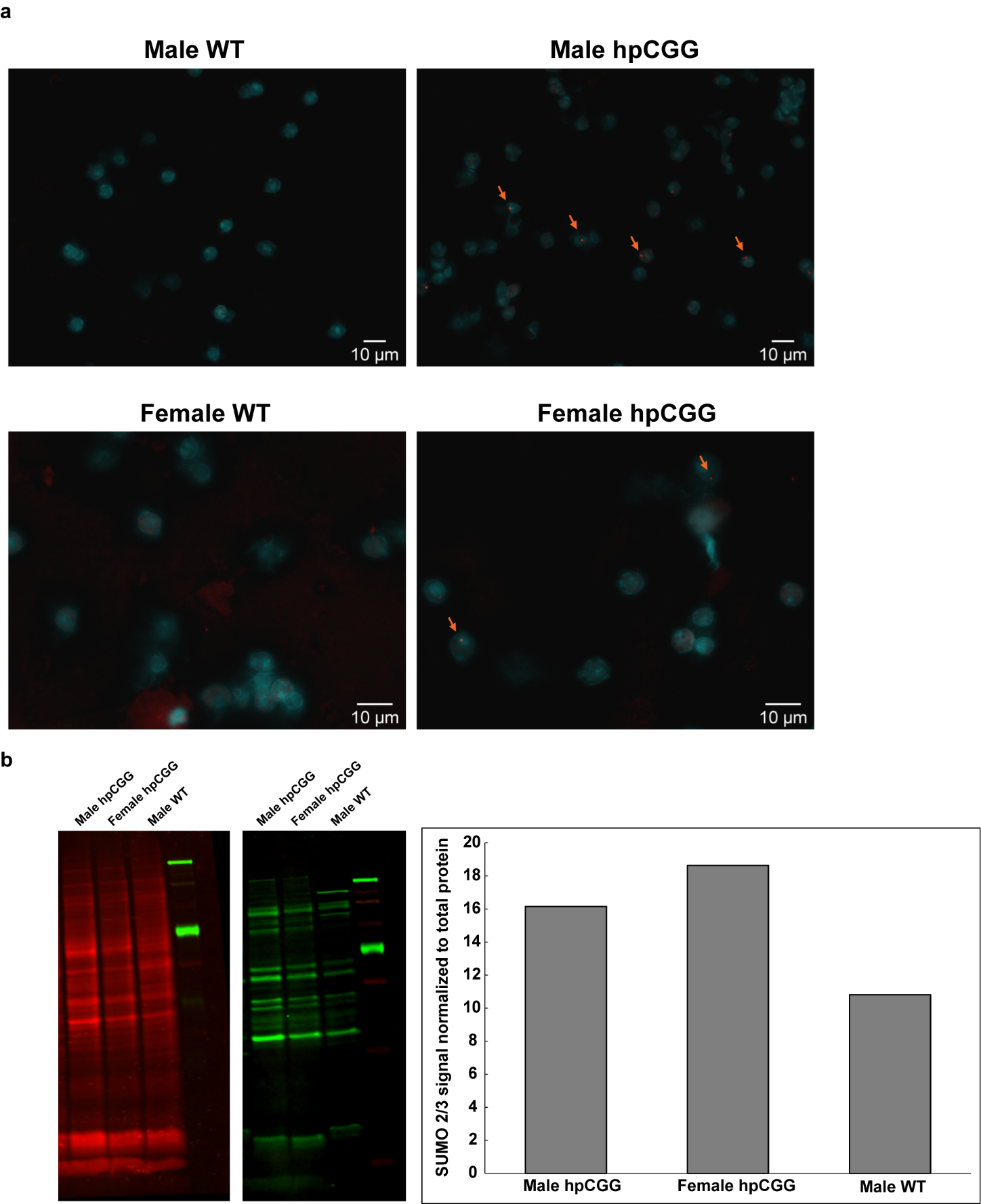
**

**Fig S6:**


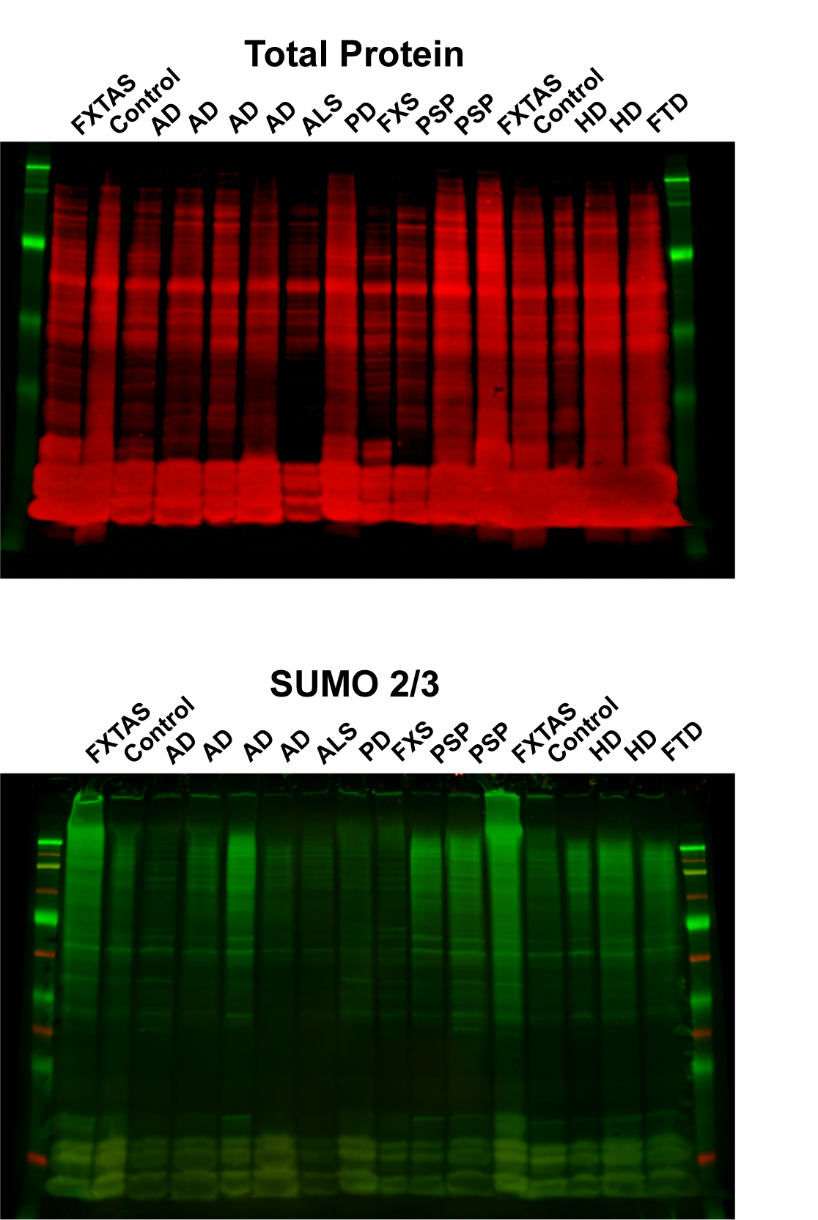


**Fig. S7:**


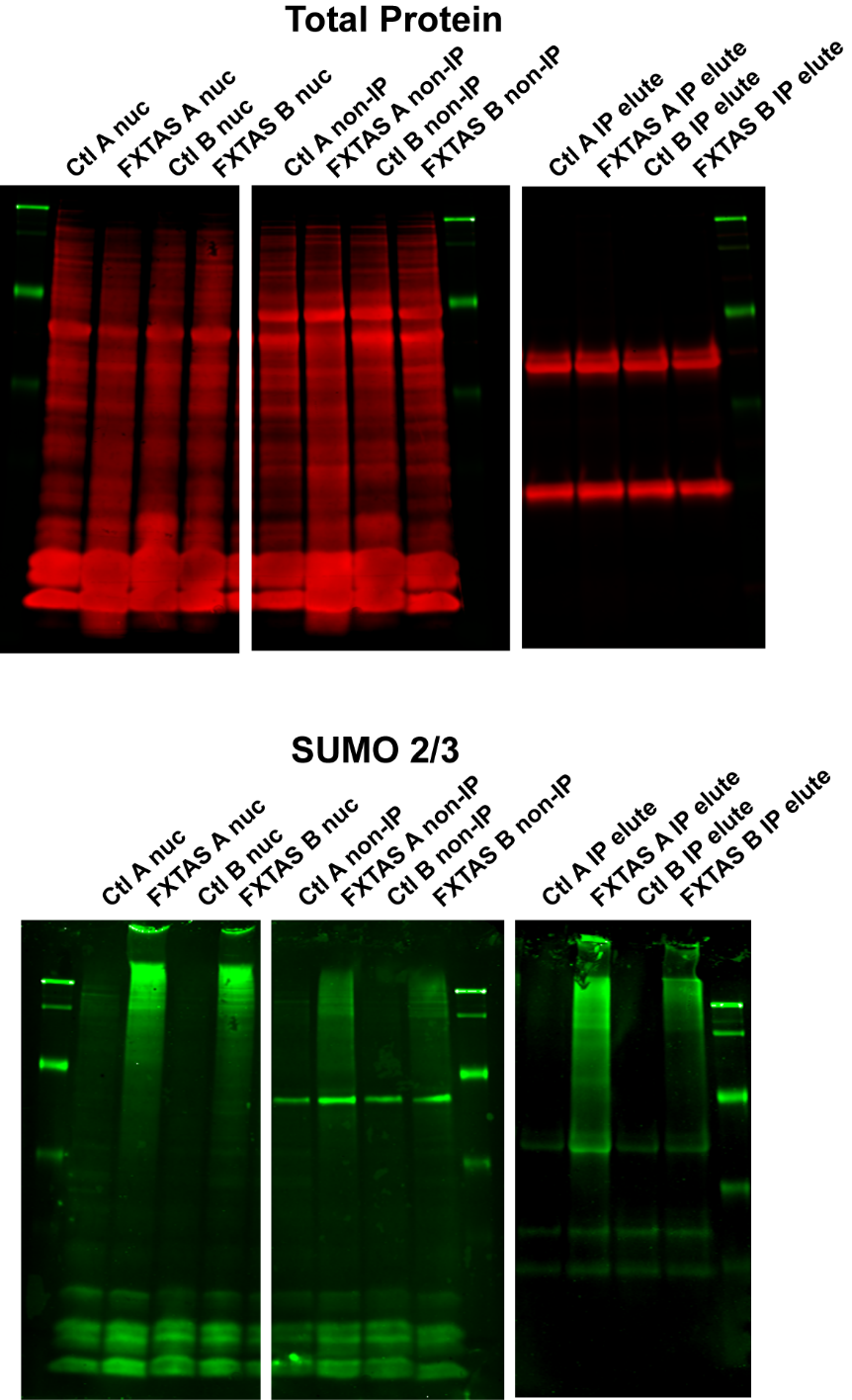


**Fig. S8:**

**
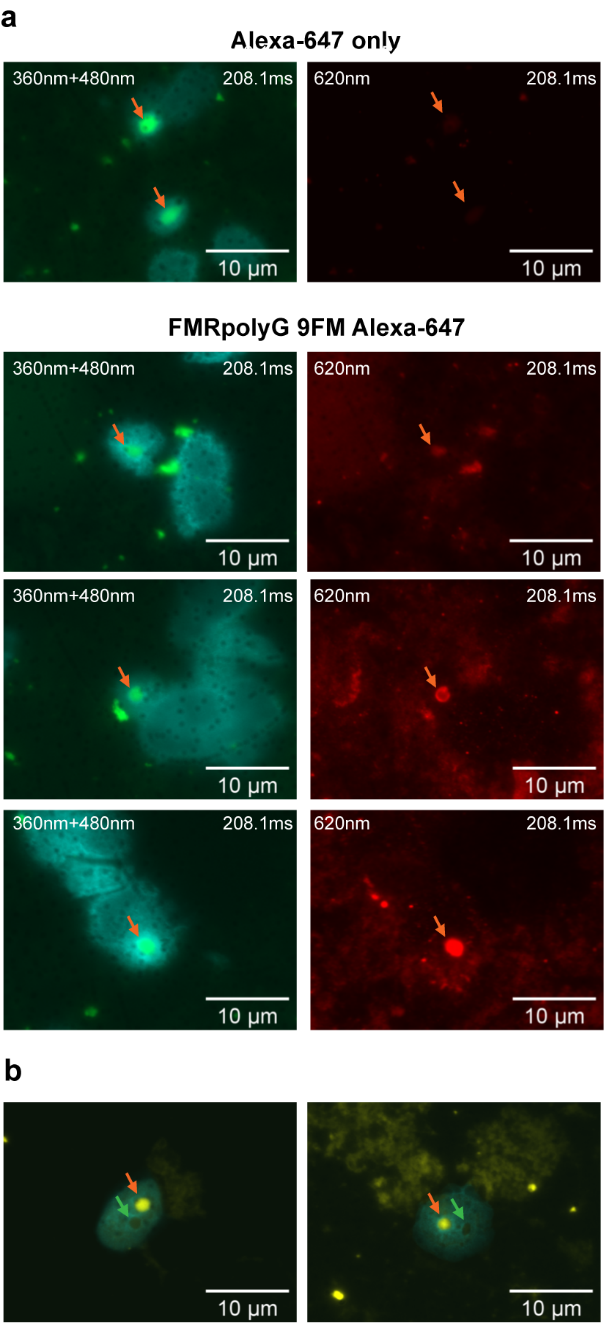
**

**Fig. S9:**


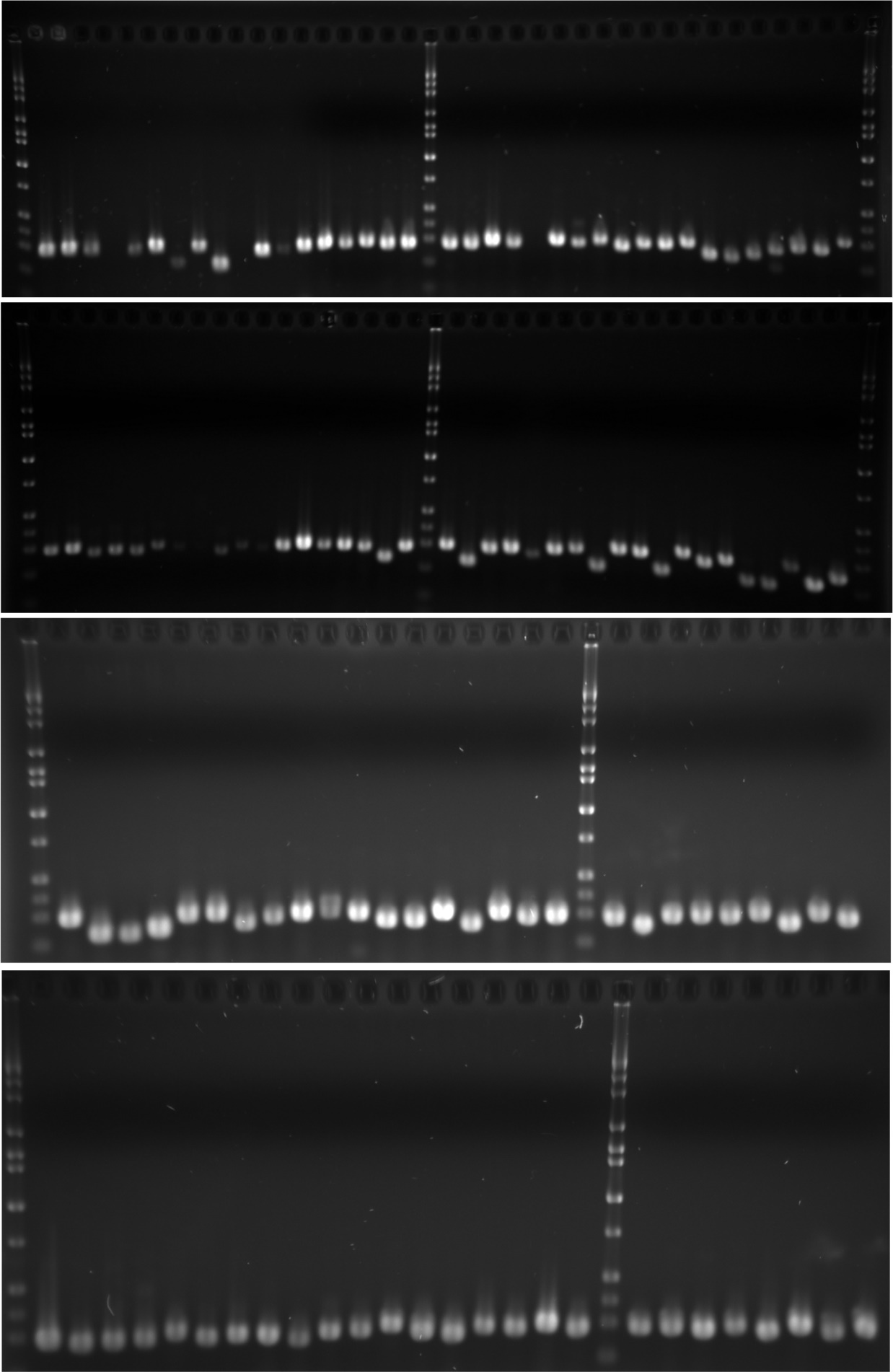


**Supplementary tables**

**Table S1:**

| **Case** | **Source** | **Diagnosis** | **Age (yr)** | **Sex** | **PMI (hr)** | **CGG** |
| --- | --- | --- | --- | --- | --- | --- |
| **B1** | UCD BR | FXTAS | 75 | M | N/A | 85 |
| **B2** | UCD BR | FXTAS | 65 | M | 30 | 105 |
| **B3** | UCD BR | FXTAS | 78 | M | N/A | 106 |
| **B4** | UCD BR | FXTAS | 69 | M | 7.5 | 98 |
| **B5** | UCD BR | FXTAS | 68 | M | N/A | 93 |
| **B6** | UCD BR | FXTAS | 83 | M | 127 | 86 |
| **B7** | UCD BR | Control | 65 | M | 67 | N/A |
| **B8** | UCD BR | Control | 69 | M | 132.5 | N/A |
| **B9** | UCD BR | Control | 62 | M | 84 | N/A |
| **B10** | UCD BR | FXS | 60 | M | 54.4 | 463 |
| **B11** | UCD BR | PD | 53 | M | 29 | 33 |
| **B12** | UCD BR | ALS | 37 | M | 43 | N/A |
| **B13** | NIH NBB | AD | 68 | M | 10.5 | N/A |
| **B14** | NIH NBB | AD | 70 | M | 6.2 | N/A |
| **B15** | NIH NBB | FTD | 72 | M | 18.6 | N/A |
| **B16** | NIH NBB | HD | 61 | M | 17.5 | N/A |
| **B17** | NIH NBB | HD | 65 | M | 9.1 | N/A |
| **B18** | NIH NBB | PSP | 66 | M | 11.8 | N/A |
| **B19** | NIH NBB | PSP | 72 | M | 11.2 | N/A |
| **B20** | UCD ADC | AD | 68 | F | N/A | N/A |
| **B21** | UCD ADC | AD | 68 | M | 3 | N/A |

Abbreviations: *Post-mortem* interval (PMI), UC Davis Brain Repository (UCD BR), NIH NeuroBioBank (NIH NBB), UC Davis Alzheimer’s Disease Center Biorepository (UCD ADC), Not available (N/A)

**Table S2:**

| **Case** | **Cell type** | **Diagnosis** | **Age (yr)** | **Sex** | **CGG** |
| --- | --- | --- | --- | --- | --- |
| **F1** | Fibroblasts | Control | 60 | F | 36 |
| **F2** | Fibroblasts | Control | 63 | M | 22 |
| **L1** | Lymphocytes | FXTAS | 55 | M | 82 |
| **L2** | Lymphocytes | Control | 56 | M | 33 |

**Table S3:**

| **Animal** | **Genotype** | **Age (mo)** | **Sex** |
| --- | --- | --- | --- |
| **M1** | hpCGG | 10.9 | M |
| **M2** | hpCGG | 10.9 | M |
| **M3** | hpCGG | 9.7 | F |
| **M4** | hpCGG | 9.7 | F |
| **M5** | WT | 11.9 | M |
| **M6** | WT | 10.4 | M |

Abbreviations: high premutation CGG (hpCGG), wildtype (WT)
